# Environmental drivers of low vaccine responsiveness in a lab-to-wild rodent model

**DOI:** 10.1371/journal.ppat.1013647

**Published:** 2026-07-21

**Authors:** Simon A. Babayan, Saudamini Venkatesan, Jessica L. Hall, Ewan W. Smith, Amy R. Sweeny, Amy B. Pedersen

**Affiliations:** 1 School of Biodiversity, One Health & Veterinary Medicine, University of Glasgow, Glasgow, United Kingdom; 2 Institute of Ecology and Evolution, School of Biological Sciences, University of Edinburgh, Edinburgh, United Kingdom; 3 Glasgow Centre for Virus Research, University of Glasgow, Glasgow, United Kingdom; Free University of Berlin Faculty of Biology Chemistry Pharmacology: Freie Universitat Berlin Fachbereich Biologie Chemie Pharmazie, GERMANY

## Abstract

Vaccination is the most effective way to prevent infectious diseases and safeguard public health. Yet, most new vaccines fail in late clinical trials, and even established ones often underperform in populations apart from those in which they were initially tested. This can lead to reduced vaccine responsiveness, breakthrough infections, and prevent or delay herd immunity. While the causes of vaccine hyporesponsiveness remain difficult to identify, quantify, and therefore address, numerous reports indicate a predominant role of environmental factors. This has notably been demonstrated by a reduction in the immunogenicity and efficacy of various vaccines when transitioning from urban to rural human populations. Here, we tested whether and, if so, how the environment can cause vaccine hyporesponsiveness. We hypothesised that if the leading causes of vaccine hyporesponsiveness were environmental, then environmentally driven hyporesponsiveness would be exacerbated when individuals are under nutritional stress; specifically predicting that high-quality diet supplementation would increase vaccine responsiveness. Finally, we predicted that parasitic helminth infections, which are more common in rural populations, would degrade vaccine responsiveness, e.g., due to their ability to modulate host immunity, and that anthelmintic treatment could rescue vaccine responsiveness in infected individuals. To test these hypotheses, we coupled lab and field experiments with structural causal modelling, and quantified diphtheria toxoid-specific IgG1 optical density (OD) in paired conspecific cohorts of laboratory-reared and wild wood mice (*Apodemus sylvaticus*) given a single or two doses of diphtheria toxoid vaccine formulated with alum, with and without diet supplementation. We found that anti-toxoid IgG1 OD was ∼ 47 % lower in the wild wood mice compared to the laboratory-reared population. We also demonstrated that, across both habitats (wild and lab), substantial variation in vaccine responsiveness was caused by diet. However, contrary to our predictions, this high-quality dietary supplementation resulted in lower vaccine responsiveness. Further, once the effects of habitat, diet, and sex were adjusted for, increasing helminth infection burdens negatively affected anti-toxoid IgG1 OD. Counterfactual predictions from our structural causal model suggested that targeting anthelmintic treatment at heavily infected individuals could have improved their anti-toxoid IgG1 OD responses by approximately 2 to 4-fold. Our results indicate that the wild environment and access to a high-quality diet play a dramatic role in shaping the immune system’s response to immunisation. Further, we showed that laboratory settings, even when using a genetically diverse, non-traditional model, systematically yielded higher IgG1 OD than was observed in free-living conspecifics on the same protocol. We provide a causally explicit modelling approach to quantify how habitat, diet, and parasites jointly shape anti-diphtheria toxoid IgG1 levels in a focal population, and to prioritise adjunct interventions such as anthelminitc treatment where model assumptions hold.

## Author summary

Vaccines often work less well outside tightly controlled trials, especially where infection and nutrition vary. Standard laboratory animals typically miss that heterogeneity and thus fail to capture important characteristics of intended recipient populations. We vaccinated wood mice (*Apodemus sylvaticus*) with diphtheria toxoid in the laboratory and in Scottish woodlands, with or without high-quality diet supplementation. We measured humoral responses, such as diphtheria toxoid-specific IgG1 optical density in serum (ELISA), a standard correlate of immunisation rather than a direct measure of clinical protection. Wild mice showed markedly lower vaccine responsiveness than laboratory conspecifics on the same protocol, while high-quality supplementation unexpectedly lowered IgG1 OD in both habitats. Among naturally infected animals, higher gastrointestinal nematode burdens tracked with weaker vaccine responses. Structural causal models integrated these data and simulated parasite removal before vaccination; under stated assumptions, the models predicted large gains in IgG1 OD for heavily infected individuals. This work highlights how environment and parasites shaped IgG1 levels in our experiment and illustrates causal modelling for immunisation in diverse hosts.

## Introduction

Unanticipated variability in individual innate and adaptive immune responses to vaccination can lead to a lack of protection to the targeted disease, continued pathogen transmission across the wider population, and an increased risk of the evolution of vaccine escape [1–5]. Both intrinsic and extrinsic factors may impact vaccine responsiveness, including genetics [6] and epigenetics [7], sex [8], reproductive status [9], prior infections [10–14], and diet [15–18]. Environmental context can thus have profound effects on the immune system [19–21], as for example, brief natural exposure can enhance B cell maturation and antibody production [22]. Together, these effects can have clinically important consequences, including lower vaccine responsiveness and clinical efficacy in rural or low-income settings [19,23]. Consequently, despite stringent clinical testing, the few vaccines that successfully complete phase III trials and reach global markets [24] can show reduced effectiveness in field deployment relative to trial settings, whether assessed as clinical protection or, as here, humoral correlates such as IgG titre or optical density, with heterogeneity across host genetics and urban versus rural deployment [19,25]. Ancestry and geography associate with divergent measles, BCG, and tetanus read-outs [21,26–28], and similar patterns recur across antigens [19]. In addition, helminths, which are concentrated in such populations [29,30], can systemically modulate immunity [31,32], yet links to vaccination are mixed [11]. Hence, while we have clear understanding that clinical vaccine efficacy depends on both vaccine formulation and recipient population, we still lack predictive power to identify poor responders and understand how intrinsic and extrinsic factors jointly interact with the environment to shape vaccine-induced protection.

Immunology has therefore sought more ecologically realistic models (e.g., “dirtier” housing, littermates, and wild-like microbiota [33–37]) to build immune systems with greater antigenic experience and ecological realism more representative of the true range of intended target populations. Yet, how such heterogeneity combines to affect immunity remains unclear [38,39]. Here, we paired randomised controlled lab-to-wild vaccination and diet supplementation in outbred wood mice (*Apodemus sylvaticus*) with structural causal models (SCMs) to estimate how habitat, diet, sex, and parasites affect diphtheria toxoid-specific serological IgG1 levels, a common indicator of vaccine responsiveness. In this system, high-quality supplementation previously cut *Heligmosomoides polygyrus* burdens and improved anthelmintic efficacy [40]. We assessed whether wild habitat lowers DTV-specific IgG1 versus laboratory settings, whether supplementation raises it, whether helminth burdens suppress it among infected animals, and, via SCM-based interventional simulation (*do*(*P* = 0)) and unit-level counterfactual prediction, how mass anthelmintic pretreatment would shift IgG1 OD at the experimental endpoint. The SCM [41] encodes biologically plausible hypotheses for adjustment under confounding, sampling, and transportability challenges [42,43] when “moving” vaccination from laboratory to field. This approach aims to bridge vaccine development from better controlled environments to an ecologically realistic yet testable and predictable mechanistic problem, quantifying environment-limited responsiveness and indicating where adjunct interventions such as dietary supplementation and deworming are most likely to yield real-world gains.

## Results

### Vaccination induced lower diphtheria toxoid-specific IgG1 OD in the wild than in the laboratory

**Fig 1:**
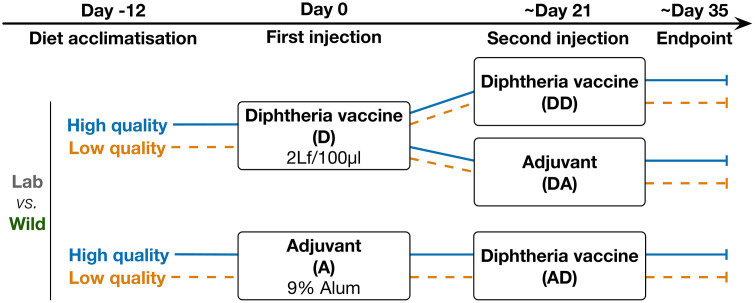
Experimental design. Wood mice from a wild-derived, now laboratory-reared, outbred colony and from the wild (Scottish woodlands) were provided enriched (TransBreed) or standard (lab: normal chow; wild: no supplementation) diets for at least 12 days (d-12). Wood mice were then allocated at random to immunisation with a 2 Lf (limits of flocculation units; manufacturer potency label) per 100 μl Diphtheria Toxoid formulated with 9% (w/v) potassium alum as adjuvant (D) or with adjuvant-only control (A). Each A or D visit used one 100 μl subcutaneous injection; the adjuvant-only arm therefore received one alum injection on prime and, where applicable, a second alum-only injection on boost, mirroring injection number in DD/DA except antigen content. Twenty-one days after their first immunisation, mice that had been immunised with their first dose (D) were then given a second dose (boost) of the vaccine (DD) or just given an adjuvant-only control (DA), while mice that had received an initial adjuvant-only control (A) were all given their first dose of 2Lf/100μl Diphtheria Toxoid + 9% alum vaccine for their second immunisation (AD). Per-arm allocation in the laboratory cohorts was AD *n* = 12, DA *n* = 12, and DD *n* = 12; across repeated sampling, the ELISA dataset comprised 213 laboratory-habitat and 98 wild-habitat observations spanning A, D, AD, DA, and DD histories.

**Fig 2:**
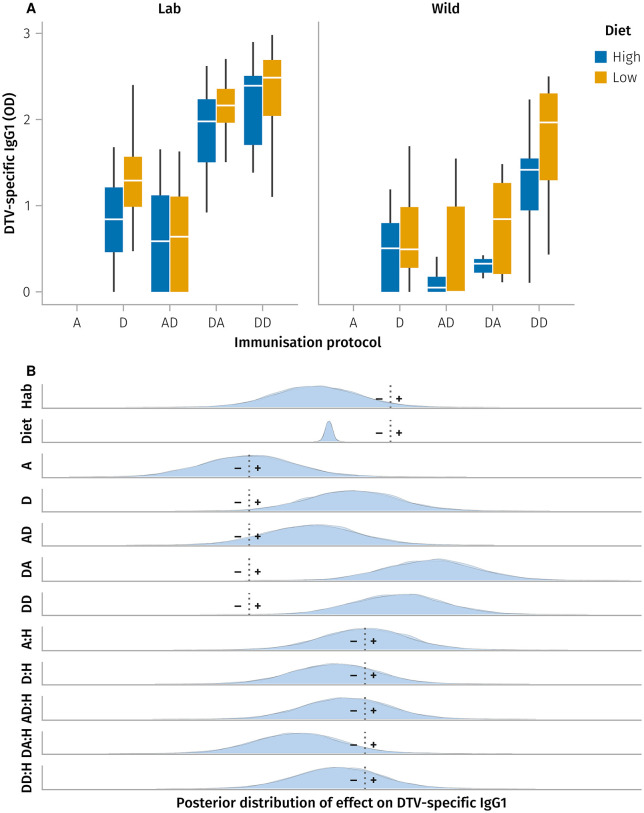
Correlates of vaccine responsiveness (DTV-specific IgG1 OD). **A**, Diphtheria toxoid vaccine (DTV)-specific IgG1 optical density (OD) in wood mice from laboratory and wild habitats provided with either high- or low-quality/control diets. Mice were immunised either once, with adjuvant-only control (A) or DTV once (D), or twice, using adjuvant alone followed by DTV (AD), DTV and subsequently adjuvant alone (DA), or two separate doses of DTV (DD). Only serum samples taken >7 days after immunisation are shown here. Box plot whiskers extend to the most extreme points within 1.5 × the interquartile range (IQR) from the box; outliers are not shown. **B**, Posterior distributions for the coefficients in the same Bayesian hierarchical model as Table 1 (Markov chain Monte Carlo, MCMC, samples). Posterior conditional distributions of the coefficients for Diet, habitat (Hab or H), and Vaccine formulation (A, D, AD, DA, DD), and interactions between each habitat and vaccine formulation (A:H, D:H, AD:H, DA:H, DD:H). Each density curve represents the distribution of 3,000 samples from one of four MCMC chains. Plus and minus signs flag whether the bulk of each posterior lies above or below zero (direction of the coefficient relative to the reference level). The dotted line marks zero on the coefficient scale (reference levels: control diet, laboratory habitat, and adjuvant-only arm A). The horizontal axis is the same coefficient scale as panel legend categories; IgG1 outcomes are OD units in panel **A.**

Wild and laboratory wood mice (*Apodemus sylvaticus*) were fed high- or low-quality diets and randomly assigned to immunisation arms A, D, AD, DA, or DD (Fig 1). Arm A received alum-only injections on the antigen + alum schedule; D one alum + diphtheria toxoid dose; DD two toxoid doses; DA toxoid followed by an alum-only boost; and AD an alum-only prime followed by toxoid, allowing us to separate adjuvant from antigen effects. Laboratory cohorts comprised 72 mice in two blocks of 36; across repeated sampling, the ELISA dataset comprised 213 laboratory and 98 wild observations (Fig 1; see “Materials, Methods, and Models”, with exact *n* per arm in Table B in S1 Appendix.) DTV-specific IgG1 was measured by ELISA from samples taken 7–35 days after the most recent immunisation, using IgG1 OD endpoint *E* as the measure of vaccine responsiveness (Fig 2, *n* = 222 ELISA observations: 165 laboratory and 57 wild). Because wild mice could not be recaptured on fixed days, we restricted the primary IgG1 analysis to this window and treated the temporal trajectories as context (Fig A in S1 Appendix; see S1 Appendix “Temporal Dynamics Analysis”). Across that window, the IgG1 OD trajectories showed broadly similar timing between habitats, with habitat differences expressed primarily as response magnitude (Fig A in S1 Appendix).

Anti-toxoid IgG1 OD was 46.9% ± 0.1% lower in the wild wood mice than their lab-based wood mouse conspecifics, regardless of the vaccine regimen given or diet (Fig 2A and 2B ‘Hab’), with model diagnostics supporting stable inference (Fig Ca in S1 Appendix). While immunisation was most effective (as measured by IgG1 OD) with two toxoid doses across both lab and wild habitats, laboratory wood mice showed little additional gain from a second toxoid dose (Fig 2A; lab DD vs D: OD = 0.2 ± 0.1), whereas the corresponding increment was larger in wild mice (wild DD vs D: OD = 1.02 ± 0.23). However, in the wild, only mice given two toxoid doses (DD) showed responses similar to laboratory-housed wood mice (Fig 2A; difference wild D vs lab D OD = 0.75 ± 0.11, wild DD vs lab DD OD = 0.61 ± 0.18; LRT of Habitat × vaccine regimen: $\Delta dof=4,\;\chi^{2}=30.6,\;p<0.001$). Only wood mice receiving the diphtheria toxoid antigen generated DTV-specific IgG1, indicating no pre-existing immunity to this antigen in any of our study populations (Fig 2A, group ‘A’). Intriguingly, dietary supplementation appeared to reduce vaccine responsiveness in both habitats, though more strongly in the wild (Fig 2B and Table 1). In the wild, supplemented animals showed relatively higher IgG1 OD early in the sampling window, so uneven recapture timing did not explain the overall negative diet association (Fig A in S1 Appendix). The apparent difference between laboratory D and AD arms in Fig 2A should likewise be interpreted in light of the fact that in arm AD, the diphtheria toxoid dose was more recent relative to blood sampling (following an adjuvant-only prime), whereas in D animals were sampled further from their single toxoid immunisation (Fig A in S1 Appendix).

**Table 1:** Posterior means and quantiles below summarise the Bayesian hierarchical model whose coefficient posteriors are shown in Fig 2B (same specification as S1 Appendix, “Multilevel Models for Treatment Effects”). To understand how different factors influence diphtheria toxoid-specific IgG1 OD in individuals, we used a statistical model that accounts for individual differences and multiple categorical variables. The model assumes $E_{i}$ (IgG1 OD for individual *i*) follows a normal distribution with mean $\alpha_{ID[i]}+\gamma_{H[i]}+\delta_{D[i]}+\eta_{V[i]}+\theta_{\left(V\times H)[i\right]}$ and standard deviation σ. Here, $\alpha_{ID[i]}$ is the individual-specific baseline (random intercept) $\gamma_{H[i]}$, $\delta_{D[i]}$, and $\eta_{V[i]}$ are the fixed effects of habitat *H*, diet *D*, and vaccine formulation *V*, respectively, and $\theta_{\left(V\times H)[i\right]}$ represents the interaction effect between vaccine and habitat. The model captures how these factors and their interaction contribute to variation in IgG1 OD.

Parameter	Mean	Std	5%	50%	95%
**Intercept**	1.02	0.85	-0.39	1.032	2.43
**Habitat**	-0.519	0.806	-1.863	-0.513	0.795
**Diet**	-0.253	0.072	-0.373	-0.253	-0.137
**A**	0.0	0.871	-3.307	-1.892	-0.438
**D**	2.062	0.856	-1.228	0.175	1.598
**AD**	1.256	0.875	-2.061	-0.651	0.84
**DA**	3.652	0.87	0.343	1.751	3.215
**DD**	2.996	0.865	-0.304	1.091	2.534
**A:H**	0.0	0.818	-0.864	0.47	1.831
**D:H**	-0.594	0.811	-1.434	-0.125	1.223
**AD:H**	-0.371	0.823	-1.244	0.103	1.472
**DA:H**	-1.348	0.818	-2.228	-0.874	0.496
**DD:H**	-0.527	0.818	-1.389	-0.061	1.31

To further quantify how habitat (lab vs. wild), diet (supplemented vs. control), and immunisation history (A, D, AD, DA, DD) shape variation in IgG1 OD (endpoint *E*), we fitted a multilevel Bayesian model with varying intercepts for each immunisation regimen and a habitat × vaccine interaction (Fig 2B, Table 1, and S1 Appendix, “Multilevel Models for Treatment Effects” for details). Convergence and sampling diagnostics are shown in Fig D in S1 Appendix, and all parameters satisfied $\hat{R}<1.01$ with the sampling diagnostics summarised there (see “Generalised linear mixed models of the effects of experimental interventions”). Posterior coefficient densities in Fig 2B and Table 1 showed the estimated main effects of Diet and Habitat, the mean effects for each vaccine-history level (A, D, AD, DA, DD) relative to the reference arm, and the corresponding habitat-interaction terms (A:H, D:H, AD:H, DA:H, DD:H). In particular, the interaction terms quantified how vaccine-history effects differed between habitats (Fig 2B and Table 1), supporting the descriptive pattern in Fig 2A that wild-habitat responses were lower overall and that some regimens (notably DA) showed stronger wild-versus-laboratory divergence. (For an arm-level descriptive summary of IgG1 OD by immunisation history × habitat × diet, see Table B in S1 Appendix.)

However, this model did not explain how habitat and diet drove poor vaccine responsiveness. Further, it did not account for potential confounding and mediating effects of mouse sex, reproductive status, body mass, body fat, or gastrointestinal parasite burdens, which we hypothesised also contributed to variation in IgG1 OD (endpoint *E*). To address these limitations, we constructed a structural causal model (SCM), denoted ${\mathbb{C}}_{VE}$, for the processes generating variation in this endpoint in this system (see S1 Appendix, “Model Construction and Validation”).

### Structural causal model: hypothesis testing and estimation

**Fig 3:**
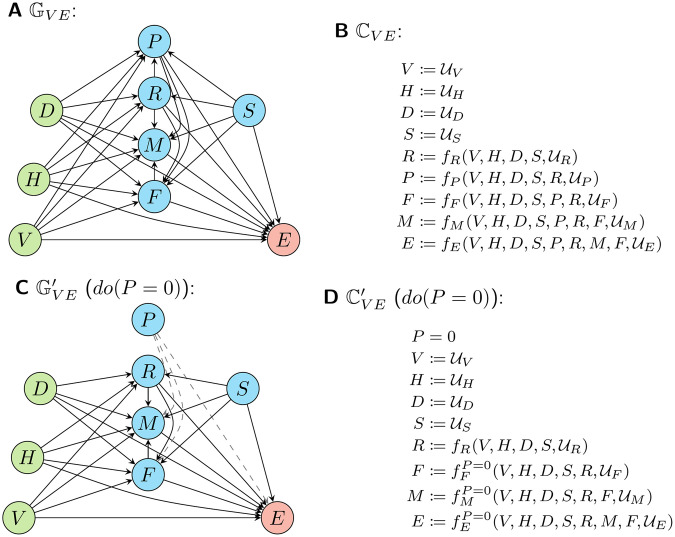
Causal model of vaccine responsiveness. Structural causal models comprise a causal graph (A, C) and an associated set of structural equations (B, D). **A**, Directed acyclic graph (DAG) ${𝔾}_{VE}$ representing hypothesised causal effects driving variation in IgG1 OD (endpoint *E*) for vaccine *V* in laboratory or wild wood mice *H*, under supplemented or control diet *D* (green nodes depict experimental treatments). Covariates, mediators, and confounders (blue nodes) included gastrointestinal parasite infection *P*, reproductive status *R*, body mass *M*, fat scores *F*, and sex ***S***. **B**, Structural causal model ${\mathbb{C}}_{VE}$ derived from ${𝔾}_{VE}$. **C**, Modified DAG ${𝔾}_{VE}^{\prime }$ representing the simulated anthelmintic intervention *do*(*P* = 0). **D**, Structural causal model ${\mathbb{C}}_{VE}^{\prime }$ under the intervention, where *P* is set to 0 and its incoming causal paths are removed.

We treated the directed acyclic graph (DAG) in Fig 3 as a mechanistic hypothesis about how habitat, diet, vaccination, and natural covariates jointly shape IgG1 OD. Validation combined (i) *Markov* implications as randomisation/balance checks (all six pairwise marginal independencies among the exogenous nodes *D*, *H*, *V*, and *S*) with (ii) supplementary mixed-model screens (see “Model validation”) for residual *V*–mediator alignment after adjustment, complementing those Markov checks under the explicit V→F, V→M, V→R, and V→P edges, before we estimated direct and total effects.

We tested the causal hypotheses encoded in DAG ${𝔾}_{VE}$ against the observed data (Fig 3; see “Model construction” methods and S1 Appendix, “Model Construction and Validation”). For example, under experimental randomisation of habitat and vaccine schedule, ${𝔾}_{VE}$ (Fig 3A) implies that vaccination assignment *V* is marginally independent of habitat *H* (H⫫V): there is no edge H→V, and every undirected path between *H* and *V* passes through a collider at a shared descendant (i.e., *E*, *F*, *M*, *P*, or *R*), so those paths are blocked marginally. Accordingly, we fitted a Bernoulli generalised linear mixed model with vaccination indicator as the response, habitat *H* as predictor, and random intercepts for mouse ID and immunisation history (as in “Model validation”); the fixed effect of *H* was compatible with no association (p≈0.71, two-sided Wald test). We repeated this pattern for the full validation battery in “Model validation”, *i.e.,* marginal balance relations among *D*, *H*, *V*, and *S* plus the auxiliary *V*–mediator screens, using them as diagnostic checks rather than a data-driven search over alternative graphs, and proceeded with the parsimonious SCM that was compatible with these tests and with biological plausibility. The resulting causal graph ${𝔾}_{VE}$ and structural equations ${\mathbb{C}}_{VE}$ are shown in Fig 3.

**Fig 4:**
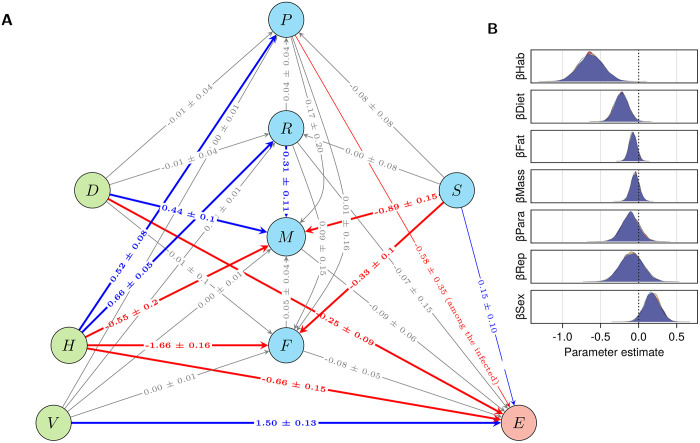
Fully resolved causal model of vaccine responsiveness (IgG1 OD). **A**, Directed acyclic graph representing point estimates (mean ± SD) of positive (blue), negative (red), and null (grey) direct causal effect distributions of Habitat *H*, Diet *D*, Fat scores *F*, body mass *M*, reproductive status *R*, sex *S*, and parasite burden *P* on IgG1 OD (endpoint ***E*)**. Edge width is proportional to the magnitude of the estimated direct effect (standardised for display). **B**, Posterior distribution for the direct effect of wild habitat *H* on IgG1 OD (endpoint *E*) in the fitted structural causal model (see Model parameterisation, interventional simulation, and counterfactual prediction).

From that DAG we fitted ${\mathbb{C}}_{VE}$ as Bayesian hierarchical linear models to estimate total and direct effects on IgG1 OD (Fig 4; Panel B gives the direct H→E posterior; see S1 Appendix, “Structural Causal Model Equations”). All SCM fits showed excellent Markov chain Monte Carlo (MCMC) behaviour with $\hat{R}<1.01$ (S1 Appendix, “Model Validation”; Fig E in S1 Appendix). For the observational (with parasites) and post-interventional (*do*(*P* = 0)) generative models used in the anthelmintic simulations, bulk and tail effective sample sizes were large for the fixed effects (typically well above 7,000 and 5,000 respectively), with the lowest bulk ESS for the residual scale σ still exceeding 3,000 in both fits (Table A in S1 Appendix). Antigen-containing regimens had a posterior mean causal effect of 1.50±0.13 on the standardised $\log_{10}(1+OD)$ endpoint *E* (see Methods) relative to adjuvant-only control arms with no diphtheria toxoid, and its expression on the IgG1 scale was strongly shaped by wild habitat and dietary supplementation. Body fat *F*, body mass *M*, and reproductive status *R* responded to diet and habitat (Fig 4A: D,H→F,M,R paths), but direct F→E, M→E, and R→E links were all null on the standardised endpoint *E*, so habitat and diet effects did not propagate to IgG1 OD through those mediators despite their association with them.

### Causal effects of habitat context, diet, sex, and parasites on IgG1 OD

**Habitat, reproduction, and natural infection.** Wood mice in the wild, unlike those in the lab, could reproduce freely and were naturally exposed to parasites. Among wild-caught individuals, 66% were classified as reproductive (R) and 52% carried adult *Heligmosomoides polygyrus* (P). Living in the wild carried an overall negative effect on vaccine-specific IgG1 of -0.46 ± 0.24 *logOD* (∼ 40% lower responsiveness) and a direct effect of -0.66 ± 0.15 *logOD* after adjusting for mediators (Fig 4A).

**Diet.** The total causal effect of high-quality diet supplementation on vaccine responsiveness was -0.25 ± 0.09 *logOD* (adjusted for habitat), i.e., roughly 22% lower responsiveness than under normal diet. The direct effect was -0.23 ± 0.09 *logOD* when further adjusting for body mass, fat, habitat, sex, reproductive status, parasite burden, and vaccine formulation, so the diet signal was largely direct or carried by unobserved pathways along D→E (e.g., immune or microbiome routes), not via mass, fat, reproduction, or parasites. Supplementation increased body mass by 2.53 ± 0.3 g on average without affecting fat scores.

**Sex.** The total causal effect of sex on DTV-specific IgG1 OD *E* indicated greater vaccine responsiveness in females, with approximately an 82% increase in vaccine responsiveness compared to males (0.26 ± 0.15 *logOD*). In our fitted SCM, this difference was predominantly attributable to the direct S→E pathway (Fig 4); although males were, on average, both heavier and fatter, the estimated direct effects of body mass and body fat on *E* were null in this model (see M→E and F→E coefficients), so we did not interpret the sex effect on IgG1 OD as being transmitted through *M* or *F* in these data.

**Parasites.** When considering the entire wood mouse population, including uninfected individuals, *H. polygyrus* burden had no clear effect on vaccine responsiveness (-0.13 ± 0.11 *logOD*). However, because parasite burdens (measured here as the number of adult worms) were overdispersed and zero-inflated (variance/mean ratio = 86.3 for *H. polygyrus*), we also estimated the causal effects of infection burden on DT vaccine responsiveness only among infected mice (Fig F in S1 Appendix). Parasite burdens had a strong negative effect on vaccine responsiveness among the infected, with a slope of -0.58 ± 0.35 *logOD* (i.e., the regression coefficient on the $\log_{10}(1+OD)$-transformed outcome scale), after adjusting for diet, sex, reproductive status, and vaccination. This negative association between parasite infection and vaccine responsiveness was also evident in the raw data analysis (Fig Cb in S1 Appendix), with a sex-stratified version shown in Fig Cc in S1 Appendix, and indicated that higher parasite burdens were associated with a significant decrease (approx. 74% decrease per 10x parasite count increase) in vaccine responsiveness. Zero-inflated negative binomial models confirmed the robustness of this relationship whilst properly accounting for the ecological reality of uninfected versus infected animals (see S1 Appendix, “Zero-Inflation Modelling for Parasite Data”).

### Anthelmintic projections: interventional simulation and counterfactual prediction

To evaluate the potential benefits of anthelmintic treatment for improving diphtheria toxoid vaccine responsiveness, we used Bayesian generative models to simulate an interventional scenario in which all mice received anthelmintic pretreatment prior to vaccination. This approach enabled us to predict individual-level IgG1 OD (*E*) under both the observed conditions (with parasites/no drug treatment) and the hypothetical intervention scenario (without parasites due to drug treatment), whilst propagating posterior uncertainty in the fitted structural model (identifying assumptions in the Methods).

We aimed to (i) quantify the population-wide improvement in vaccine responsiveness after complete parasite elimination, (ii) identify which individuals would benefit most from anthelmintic drug treatment, and (iii) assess the magnitude of the effects of parasite removal in each sex and reproductive cohort. Using posterior predictive sampling from our Bayesian models, we generated paired counterfactual IgG1 OD predictions that maintained each individual’s unique characteristics while removing the effects of parasite infection. Fig 3C and 3D illustrate the structure and equations of the model under the simulated anthelmintic intervention, where all incoming edges to *P* are blocked and *P* is set to zero.

**Fig 5:**
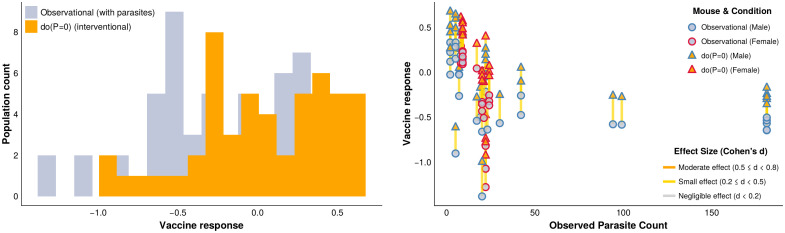
Effect of simulated population-wide anthelmintic drug treatment on vaccine responsiveness (diphtheria toxoid-specific IgG1 OD; endpoint *E*). **A**, Population-wide *marginal* posterior predictive distributions for infected mice: observational branch $E_{factual}$ (with parasites; light grey) versus interventional posterior predictive $E_{do(P=0)}$ under simulated parasite elimination on *E* (orange). The rightward shift indicates improved IgG1 OD at the endpoint when the parasite pathway into *E* is removed in the fitted structural model. **B**, *Counterfactual* (paired, within-mouse) layer: posterior predictive summaries $E_{factual}$ (circles) and $E_{do(P=0)}$ (triangles) for the same individuals and random effects, plotted against observed parasite burden (males, blue outlines; females, red outlines). Vertical segments connect paired draws, i.e., realisations of $\Delta E=E_{do(P=0)}-E_{factual}$; line colour encodes clinical significance of ΔE via Cohen’s ***d*.**

Our Bayesian models predicted two distinct but complementary effects of parasite elimination prior to vaccination. At the population level, the mixed-model coefficient for vaccination $\beta_{V}$ comparing post-interventional to observational predictions would have improved by 74.3% relative to the population mean among infected individuals (Fig 5A), representing the enhancement in vaccine responsiveness across the entire population. At the individual level, parasite elimination would have produced a predicted mean increase of 108.1% in vaccine responsiveness per mouse compared to their own individual baseline responses (i.e., with their actual parasite counts). These individual-level benefits varied considerably, ranging from 13.2% to 298.8% improvement (Cohen’s d mean = 0.20 ± 0.03).

Further, the magnitude of the predicted improvement in vaccine responsiveness was not strictly proportional to the observed parasite burdens (Fig 5B), with sex and reproductive status contributing to the effect of parasite elimination on vaccine responsiveness (Table 2).

**Table 2:** Summary of the linear mixed model estimating the individual-level effect sizes (Cohen’s d) of anthelmintic treatment on predicted changes in diphtheria toxoid-specific IgG1 OD (endpoint *E*). The model includes fixed effects for Diet (*D*), Reproductive status (*R*), Sex (*S*), Vaccination status (*V*), Body mass (*M*), Fat scores $\left(\dot{F}\right)$, and the interaction between Sex and Reproductive status (S×R). Coefficients (Coef.), standard errors, z-values, and p-values (Pr(>|*z*|)) are reported for each effect. This table quantifies the predictors of variation in IgG1 OD improvements following parasite elimination, highlighting significant contributions of reproductive status, vaccination, body mass, and a marginal interaction between sex and reproductive status.

Effect	Coef.	Std. Error	z-value	Pr(>|z|)
D	0.01899	0.01063	1.79	0.0742
R	0.06754	0.02191	3.08	0.0021
S	0.05167	0.03221	1.60	0.1087
V	0.03123	0.01448	2.16	0.0310
M	-0.01906	0.00848	-2.25	0.0247
$\dot{F}$	0.00747	0.00702	1.07	0.2868
S × R	-0.03216	0.01771	-1.82	0.0695

## Discussion

Using lab-to-wild wood mice and structural causal modelling, we showed how intrinsic and environmental factors jointly shaped diphtheria toxoid-specific IgG1 OD. Wild mice averaged 46.9 ± 0.1% lower IgG1 OD than laboratory counterparts under the same protocol in a cleaner setting; contrary to prediction, high-quality supplementation lowered IgG1 OD in both habitats. Among infected animals, higher *Heligmosomoides polygyrus* burdens tracked with weaker vaccine-specific responses. Habitat, diet, sex, and parasites should therefore be considered together when interpreting IgG1 read-outs, paralleling human evidence [21,44–46].

Wild mice remained hyporesponsive relative to laboratory conspecifics even after boosting immunisations: sampling between 7 and 35 days post-immunisation captured primary and secondary kinetics, with median peaks near 22 days in both habitats but lower magnitudes in the wild. The environment thus appeared to attenuate IgG1 amplitude more than timing, with possible implications for clinical efficacy and duration of protection (not measured here), a pattern consistent with phase III setbacks and rural–urban gaps in human programmes [19,24,47]. While we used alum (typically promoting type-2 reposnes), the habitat and diet differences we observed motivate trials of adjuvants that favour more polarised Type 1 activity, even for infections where Type 2 responses are desirable or expected, as in chronic helminth infections.

*H. polygyrus* mediated part of the wild reduction in responsiveness: among infected animals, higher burdens were associated with lower IgG1 OD, aligning with many reports in helminth infections [10,48–51] but not all [11,13,52]. These conflicting reports may reflect different approaches to conditioning on infection and burden scales: we saw a negative slope only among infected animals. Anthelmintic co-intervention could therefore improve population vaccine responsiveness where parasitic helminth prevalence and intensity are high. Models here suggested that reproductive females in particular gained most from deworming. Environmental suppression of vaccine immunogenicity in free-living hosts, together with known rural helminth burdens in people, are consistent with the hypothesis that human populations may show wider response heterogeneity than laboratory-based estimates suggest.

Our read-out was limited to diphtheria toxoid-specific IgG1 (OD), so the data constrain intermediate causes more sharply than molecular pathways. The strong negative association between adult *H. polygyrus* burdens and IgG1 OD in infected mice is compatible with each of the usual mechanistic accounts, including Th2-biased counter-regulation limiting class-switched output (as we assay it) [53,54], active parasite modulation of innate and adaptive compartments [32,55], or immunological divergence between naturally acquired wild infections and laboratory-dominant strains [56]; these explanations are not mutually exclusive. At the same time, the fitted SCM retained a substantial direct negative effect of wild habitat on *E* after conditioning on measured burdens, reproduction, mass, and fat, so adult worm counts alone do not account for the full laboratory–wild contrast in toxoid-specific IgG1 production. Additional factors might include co-circulating pathogens, nutritional and microbiota ecology, and stable host-intrinsic differences in responsiveness that our design does not identify. Separating those possibilities requires immune profiling before infection and vaccination [57,58]. Wood mouse herpesvirus (WMHV) is common (about 10–70%) and concentrates in heavy, reproductively active males, whereas WMHV-positive adults are recaptured less often, together tracing demographic risk and a likely fitness cost of infection [59]. Nematode coinfection elevates WMHV acquisition in a burden-dependent manner, anthelmintic treatment lowers WMHV odds, while improved nutrition mitigates helminth-driven increases in viral susceptibility in paired lab–field experiments on the same host system [60], paralleling CMV-associated dampening of human SARS-CoV-2 vaccine responses [14]. For intestinal coccidia, experimental coinfection of *Eimeria hungaryensis* with *H. polygyrus* lowers peak oocyst shedding while delaying worm expulsion and prolonging nematode egg output [61]; in the field, anthelmintic-induced nematode suppression transiently raises *Eimeria* abundance [62]; and nematode-specific IgG1 can correlate positively with worm burden in juveniles but negatively in adults [63]. Future studies could add WMHV and intestinal *Eimeria* alongside nematode burden to test how much variance in toxoid IgG1 *E* they explain beyond worms alone and whether the direct wild H→E estimate shrinks.

Diet effects ran counter to expectation: supplementation lowered diphtheria toxoid-specific IgG1 OD in both habitats. Repeated bleeds showed supplemented wild mice comparatively higher in IgG1 OD early in the 7–35 day post-boost window, so uneven recapture timing alone is unlikely to explain the diet penalty. Several non-exclusive mechanisms could apply, including Th2- or repair-biased reallocation [53,54]; metabolic or microbiome shifts; seasonal energy surplus, chow-specific microbiota, or alum–metabolism interactions. The same dietary supplement had previously reduced worm burdens and improved anthelmintic effectiveness [40]; we did not detect lower burdens here, which may be attributable to differing seasonal exposure or nutrition–parasite coupling between studies. Females maintained higher IgG1 OD than males, consistent with widespread reports of sex dimorphism [8,64–70]. In the fitted structural model, this difference was predominantly direct (S→E), with null estimated mediating effects of body mass and fat on IgG1 OD.

Bayesian predictions under *do*(*P* = 0) estimated a 74.3% improvement in the vaccination coefficient on IgG1 OD among *H. polygyrus*-positive mice but only 4.3% population-wide, illustrating how zero-inflated, overdispersed worm burdens can mask real anthelmintic benefit in mean population summaries while concentrating gains in the infected tail; the 20-fold spread in predicted individual responses therefore supports measuring burdens and targeting deworming [10,49–51]. In schoolchildren from Ugandan schistosomiasis-endemic islands, intensive praziquantel administration did not uniformly boost responses to unrelated vaccines in the POPVAC-A trial [71]. Interpreting those contrasts requires the identifying assumptions, positivity considerations under sparse infection, and the sensitivity battery laid out under *Structural causal models* (Materials, Methods, and Models); additional translational limits include imperfect anthelmintic efficacy under realistic dosing, extension beyond a wood-mouse IgG1 correlate without affinity or neutralisation assays [19], and limited resolution of microbiota and nutrient pathways. Wild mice likely exceed the laboratory colony in neutral diversity, which could add to habitat effects without genotyping to separate them. Fewer commercial immunological reagents exist for wood mice than for common inbred strains, so cytokine-level mechanisms would require bespoke transcriptomic or proteomic work beyond this study [72]. Paired randomised laboratory–field deployment partly offsets these caveats relative to observational wildlife work alone. Habitat and diet were randomised, vaccination and sampling followed the same schedule in laboratory and wild cohorts, and ELISA used one protocol, which limits settlement and access biases that often affect trap-based field studies. Wild mice still experienced natural worms, seasonal food, and reproduction that conventional laboratory housing largely removes, so we retain realistic infection and demography while retaining the laboratory versus free-living comparison beyond a cage-only experiment.

In conclusion, grounded in that paired randomised laboratory–field contrast, explicit causal graphs and Bayesian SCMs [39] offer a practical route to prioritise interventions (diet, deworming, adjuvant choice) when environmental heterogeneity is large, while quantifying how far standard laboratory housing can inflate measures of vaccine responsiveness relative to free-living individuals under the same protocol. Vaccine development should therefore stress-test correlates under realistic ecology, not only optimise them in conventional populations.

## Materials, methods, and models

### Ethics statement

All animal work was conducted in compliance with the UK Animals (Scientific Procedures) Act 1986. All laboratory and field experiments were approved by the University of Edinburgh Ethical Review Committee and carried out under the Project Licence 70/8543. The dosage of diphtheria vaccine was tested in laboratory-bred wood mice for safety before the experiments. Animal sacrifice was performed using appropriate Schedule 1 Methods. Fieldwork was carried out with permission of the Forestry Commission Scotland under the permit SUR09.

### Vaccine formulation and administration

The vaccine used in this study was a commercially available diphtheria inactivated toxoid (DT, Alpha Diagnostic International). The vaccine, DTV henceforth, was prepared in our laboratory one day before administration, using 2 Lf (limits of flocculation units per manufacturer labelling) of vaccine-grade DT protein formulated with alum as the adjuvant (9% (w/v) potassium alum in water), following a standard alum-based sensitisation approach [73]. The vaccine was administered subcutaneously in a volume of 100μl. The control group was injected with 100μl of the corresponding alum-only preparation in 1X PBS.

### Laboratory wood mouse experiments

#### Wood mouse colony and housing.

The laboratory experiments were carried out on a colony of wild-derived but now captive outbred wood mice bred and maintained at the University of Edinburgh [40]. The laboratory wood mouse colony has been maintained as an outbred colony for over 10 generations. Mice were housed in conventional laboratory conditions with controlled temperature (20–22 °C), humidity (45–65%), and 12:12 light-dark cycle. Standard husbandry followed ASPA guidelines with enrichment including nesting material and shelter. All laboratory wood mice used in experiments were 8–16 weeks old and were sexually mature, but reproductively naive.

#### Diets.

Two commercial laboratory diets were used in this study. TransBreed (SDS Diets Ltd., UK) is a high-quality breeding diet containing 20.1% crude protein, 10.1% crude fat, 3.5% crude fibre, and 4.9% crude ash, with enhanced nutritional content optimised for reproductive performance and immune function. We have previously shown that wood mice supplemented with TransBreed are more resistant to the gastrointestinal nematode *Heligmosomoides polygyrus*, cleared worms more effectively after anthelmintic treatment, and produced higher titre general (total IgA) and parasite-specific (IgG) immune responses in both wild and laboratory conditions [40]. The other half of the mice in this experiment were fed RM1. RM1 (Rat and Mouse Maintenance 1, SDS Diets Ltd., UK) is a standard maintenance diet containing 14.4% crude protein, 2.7% crude oil, 4.7% crude fibre, and 6.0% crude ash. TransBreed provides substantially higher energy density (20.1% vs 14.4% protein; 10.1% vs 2.7% fat) and enhanced micronutrient content compared to RM1, including elevated levels of vitamins, minerals, and essential fatty acids that support improved immune function and reproductive performance. Both diets were used in the laboratory colony, but only TransBreed was used for supplementation in the wild experiment.

#### Vaccination regime.

The laboratory experiment was conducted in two replicate blocks, with 36 animals in each block (18 males, 18 females). At 12 days before the start of the experiment (d-12), all mice were shifted to new diet regimes and given time to acclimatise; half of the animals in each block (9 males and 9 females) were given TransBreed. Within each diet regime, both male and female mice were randomly assigned to one of three experimental groups (Fig 1). On d0, animals belonging to Groups ‘DD’ & ‘DA’ (n = 24) were subcutaneously injected with 100μl of DTV, and Group ‘AD’ (n = 12) were injected with the adjuvant control. Small blood samples were taken via tail snip on d14 and d17 to measure the primary antibody response. Twenty-one days (d21) after the first injections, the animals of Group ‘DD’ (n = 12) were injected again with 100μl of DTV (‘DD’: diphtheria vaccine followed by diphtheria booster), while those assigned to Group ‘DA’ (n = 12) were injected with the control dose as described above (‘DA’: DTV followed by adjuvant only). Animals assigned to Group ‘AD’ (n = 12) that had previously been injected only with the control adjuvant only dose now received an injection of 100μl DTV (‘AD’: adjuvant followed by DTV). Another blood sample (20–50μl) was taken a day later (d22) by cheek venipuncture. All animals were sacrificed on d35 (i.e., 14 days after the second injection) and a blood sample was taken for measuring the antibody response. The same procedures and timeline were carried out for the second experimental block of animals (n = 36). Mice were co-housed throughout the experiment, in same-sex groups of three, with equal representation of the three experimental groups in each cage to prevent confounding cage effects.

### Wild wood mice experiment

We conducted a 10-week field experiment in a natural population of wood mice with a design similar to that of the laboratory experiment described above. The experiment was conducted in a woodland in Falkirk, Scotland, UK (Callendar Wood, 55.990470, -3.766636), where we established four trapping grids (60m × 40m per grid) with 10m spacing between each of the 35 trapping stations per grid; neighbouring grids were separated by more than 50 m. At each station, we set a pair of Sherman live traps (H.B. Sherman 2 × 2.5 × 6.5-inch folding trap, Tallahassee, FL, USA). We randomly selected two of the four grids for dietary supplementation, initiating the treatment 12 days before live-trapping began. Specifically, 6 kg of TransBreed pellets were evenly scattered across each treated grid twice weekly (approximately 170g per trapping station), with supplementation continuing throughout the experiment. The remaining two grids served as controls and received no additional food. All wood mice had access to their natural diet, so the supplemental pellets on the treated grids provided *ad libitum* access to high-quality nutrition while allowing continued foraging for natural food sources.

From July-September 2018, wood mice were trapped 2–3 nights per week using live traps baited with grains, carrots, and bedding. Additionally, traps on the supplemented grids were also baited with 1–2 TransBreed pellets. For identification, all newly captured mice weighing > 13g were subcutaneously tagged with a unique 9-digit microchip passive induced transponder (PIT tag; FriendChip AVID2028, Norco, CA, USA). Upon first capture, mice were randomly assigned to one of the three vaccine treatment groups ‘DD’, ‘DA’, and ‘AD’, described above and in Fig 1; mice assigned to these groups received the same primary and booster doses of DTV or alum control as described above. Throughout the experiment, small volume blood samples were taken weekly via tail snip from each tagged animal to measure their antibody response to the diphtheria immunisation, on days as close to possible as their laboratory counterparts. Animals recaptured 14 or more days after their second injection were sacrificed and a terminal bleed was collected to measure their antibody response and adult *H. polygyrus* worm burdens. *H. polygyrus* is a natural gastrointestinal nematode found at high prevalence (20–100%) in wild populations of *Apodemus sylvaticus* [40,62,74,75], as well as being a well-studied model system of human gastrointestinal nematodes where it has been found to be highly immunomodulatory [55,76].

Based on cross-grid PIT tagging, three wild mice shifted grids and therefore changed their diet-assignment status during the study. Movement patterns varied among these individuals: one switched grids once and remained in the new grid, whereas the other two split their captures approximately equally between grids, showing bidirectional rather than unidirectional movement. Removing these individuals had negligible effects on mean estimates, so they were retained with their observed, time-varying diet assignment accounted for in the models (including lagged supplementation effects; see S1 Appendix, “Temporal Dynamics Analysis”).

Additionally, at all captures, we took the following demographic metrics for each individual: sex, body condition, reproductive condition, weight (g), and length (mm), and assigned juvenile versus adult status based on coat colour. Sex and reproductive condition of each mouse were assigned by examining the genitals, with males having larger urogenital gap compared to females. Males were classified as reproductively active if their testes had visibly descended to scrotal sacs, and females were classified as reproductively active if they had a perforated vagina or were visibly pregnant or lactating. Body condition was measured by assigning dorsal and pelvic fat scores on a scale from 1-5, where 1 represented a mouse with very low condition/fat reserves, while a 5 represented a mouse with ample fat reserves. This was achieved by palpating the back and pubic bones [77]. Both scores were added to provide a single metric of body condition for analysis.

### Vaccine responsiveness: DT-specific IgG1

We report vaccine responsiveness as diphtheria toxoid-specific IgG1 optical density (OD) by ELISA, which we denote the endpoint *E* in the causal models, a humoral correlate of immunisation rather than a direct measure of clinical vaccine efficacy or antibody production rate. This is a standard method used in vaccinology to measure the antibody-specific response to immunisation and is used specifically for the diphtheria toxoid vaccine used here [78]. Blood samples from both the laboratory and the wild mice were centrifuged in a standard benchtop microcentrifuge for 1.5 mL tubes at 13,000 rpm for 10 min within 4–6 hours of collection. The sera were separated from the pellets and stored at -80 °C until further analysis. 96-well plates (Nunc MicroWell) were coated overnight at 4 °C with diphtheria toxoid (2μg/mL) diluted in carbonate buffer (50 μl per well). After washing the plates with Tris-buffered saline (TBS; 10 × concentrated stock diluted to working strength) and Tween80 thrice, 100 μl of TBS(1X)-4% BSA was added per well and incubated at 37 °C for 2 hours to block non-specific binding sites. Plates were then tapped dry and 50 μl of serum samples serially diluted (starting dilution 1:100) in TBS (1X)-4% BSA buffer were added per well and left at 4 °C for binding overnight. The next day, 50 μl HRP-conjugated anti-mouse IgG1 detection antibody (Southern BioTech) per well were added after washing the plates 4 times with working-strength TBS (from the same 10 × stock diluted for use) and Tween80 and tapping them dry. After incubation at 37 °C for 1 hour, the plates were washed again, four times with working-strength TBS (from the same 10 × stock) and Tween80 and then twice with dH20. Then, 50 μl of TMB substrate solution were added per well and the enzymatic reaction was left to develop in the dark for 7 min. The reaction was stopped after 7 min using 25 μl of 1 N sulphuric acid per well. Absorbance at 450 nm was recorded using an ELISA plate reader (Multiscan, Ascent Labsystems) immediately thereafter. For each sample, blank-centred optical densities (OD) of three consecutive wells (of dilutions 1:3200, 1:6400, 1:12800) were averaged to obtain diphtheria-specific IgG antibody measurements for each individual. The same ELISA workflow was applied to wild and laboratory sera. ODs were blank-centred. We did not apply inter-plate scaling (e.g., a shared positive control or standard-curve normalisation), so residual plate effects may contribute to measurement noise; however, samples from different groups were interleaved across plates to minimise systematic bias. ODs were background-corrected by blank subtraction, and values at/below the per-plate cut-off were retained as assay-floor observations (not censored). Counts of assay-floor values by habitat and immunisation arm under the primary >7-day sampling window are reported in Table C in S1 Appendix (S1 Appendix, “Assay-floor and non-responder checks”); excluding these observations did not change the qualitative laboratory–wild contrast in mean IgG1 OD.

### Data processing and analyses

ELISA optical densities of diphtheria (DT)-specific antibodies were taken as endpoint *E* by applying a $\log_{10}(1+x)$ transform followed by Z-score standardisation. Body weight (*M*) and summed dorsal/pelvic fat scores (*F*) were Z-score transformed separately. All other variables were treated as binary. All data processing was performed in Julia v1.12.6 [79] using packages CSV.jl v0.10.16 [80] and DataFrames.jl v1.8.2 [81]. Large Language Models were used for code refactoring and optimisation. Detailed quantitative methods are available in S1 Appendix, “Bayesian Statistical Implementation”. Analysis code to reproduce the models is available at https://github.com/SimonAB/Apodemus_vaccines

#### Generalised linear mixed models of the effects of experimental interventions.

We used generalised linear mixed models (GLMMs) to estimate the main effects of habitat, diet, and vaccine formulation on vaccine-specific antibody concentrations. Mouse ID was modelled as a random effect to account for repeated measurements. Interactions between vaccine regime and habitat, and between diet and habitat, were included to test whether wild mice responded differently to treatments than laboratory mice. Likelihood ratio tests (LRT) evaluated the contribution of interaction terms. The package MixedModels.jl v5.1.0 [82] was used for all GLMMs and LRTs.

We then implemented Bayesian hierarchical models to minimise the effects of data imbalance and provide robust uncertainty quantification. Weakly informative Gaussian priors were specified for intercepts and regression coefficients. Residual standard deviations were given Exponential(1) priors, and random effect standard deviations used Exponential(1) priors with non-centred parameterisation to improve sampling efficiency. Prior predictive checks are performed to ensure biological plausibility (Fig B in S1 Appendix). Posterior estimates were sampled using Hamiltonian Monte Carlo with the No U-Turn Sampler [83], with 4 chains of 3,000 iterations each after 1,000 warmup iterations. Convergence was assessed using $\hat{R}<1.01$ for all parameters, together with the MCMC sampling diagnostics in Fig D in S1 Appendix. Turing.jl v0.44.5 [84] was used for Bayesian modelling; CairoMakie.jl v0.15.10 (Makie.jl v0.24.10) [85] was used for plotting. To address heterogeneous sampling times (especially in wild recaptures), we report a dedicated temporal-dynamics analysis (Fig A in S1 Appendix).

#### Structural Causal Models.

Structural causal modelling (SCM) [41] provides a framework for identifying and estimating causal effects when randomised and naturally varying components coexist, by explicitly representing causal assumptions through directed acyclic graphs (DAGs). Prior predictive checks validated that our weakly informative priors produced biologically plausible IgG1 OD values whilst appropriately covering the observed data space (Fig B in S1 Appendix). We used SCMs to quantify the causal pathways through which habitat, diet, and parasite infection influence vaccine responsiveness, distinguishing between direct effects (e.g., parasites directly modulating immune responses) and indirect effects mediated through body condition or reproductive status.

Our primary estimand was the *average direct causal effect of wild habitat on vaccine responsiveness* conditional on diet, sex, reproductive status, body mass, fat scores, and parasite burden: $𝔼[E^{H=1}-E^{H=0}|D,S,R,M,F,P]$, where *H* = 1 denotes wild habitat and *H* = 0 denotes laboratory habitat. Secondary estimands included the direct causal effects of diet supplementation, parasite burden, and sex on vaccine responsiveness, and the *average treatment effect of hypothetical anthelmintic intervention*: $𝔼[E^{P=0}-E^{P=observed}]$, representing the population-level improvement in vaccine responsiveness under complete parasite elimination.

Our approach relied on several key identifying assumptions, stated in line with the randomised laboratory–field design above and the DAG validation in “Model validation”.

(1) **Confounding control:** For randomised components (*V*, *D*, *H*), identification used design-based balance, checked empirically in “Model validation”. For naturally varying mediators and the endpoint (*P*, *R*, *M*, *F*, *E*), we assumed no residual confounding beyond the parents encoded in ${𝔾}_{VE}$ after conditioning on measured covariates. Co-infections, microbiota, grid-level supplementation, and other field processes omitted from the graph could still induce bias, as we acknowledge when contrasting laboratory and wild ecology.
(2) **Stable Unit Treatment Value Assumption:** We treated each animal’s read-out as determined by its own realised vaccination, diet, and habitat assignments. In the laboratory, mice were co-housed in same-sex trios with balanced representation of vaccine arms across cages (see “Laboratory wood mouse experiments”), which limits but does not completely remove cage-level spillover. Wild mice were free-living across trapping grids, so local interference, transmission, or supplementation spillover cannot be ruled out.
(3) **Consistency:** We assumed that nodes in ${𝔾}_{VE}$ map to their operational definitions in the protocol: *H* contrasts laboratory housing with the field deployment; *D* contrasts diet arms within each habitat; *V* follows the alum–toxoid schedule; and *E* is diphtheria toxoid-specific IgG1 OD by ELISA (Methods), a correlate rather than clinical protection. The intervention *do*(*P* = 0) matches the parasite-free structural model ${𝔾}_{VE}^{\prime }$ (Fig 3C - 3D), *i.e.* an idealised elimination of adult worm burden on the measured scale, not dynamic transmission or imperfect drug efficacy.
(4) **Functional form:** Structural equations were implemented as regressions with the links and scales in “Model parameterisation, interventional simulation, and counterfactual prediction” and the S1 Appendix generative-model descriptions ($\log_{10}(1+x)$-transformed *E*; *Z*-scored *M* and *F*; Bernoulli structure for binary components). This is an additive linear specification on those scales; zero-inflated parasite analyses and temporal dynamics (S1 Appendix) probe misspecification of infection intensity and sampling-time effects.
(5) **Positivity / common support:** Randomisation supports overlap for *V*, *D*, and *H*. Natural-history quantities (*P*, *R*, *M*, *F*) are unevenly supported in the data: parasite burdens are zero-inflated and overdispersed (Results; S1 Appendix, “Zero-Inflation Modelling for Parasite Data”), and wild recapture timing varies (S1 Appendix, “Temporal Dynamics Analysis”), so some contrasts lean on model-based extrapolation rather than dense empirical support alone.

To assess robustness to assumption violations, we implemented comprehensive sensitivity analyses including: (i) E-value calculations [86] to quantify the minimum strength of unmeasured confounding needed to explain away observed effects (Fig G in S1 Appendix; see S1 Appendix, “E-value Sensitivity Analysis for Unmeasured Confounding”); (ii) prior sensitivity analysis to evaluate robustness to model specification across different prior scales (see S1 Appendix, “Prior Sensitivity Analysis”); (iii) zero-inflation modelling for realistic parasite count analysis (see S1 Appendix, “Zero-Inflation Modelling for Parasite Data”); (iv) temporal dynamics analysis to characterise vaccine response kinetics (see S1 Appendix, “Temporal Dynamics Analysis”); and (v) causal assumption testing beyond standard conditional independence (see S1 Appendix, “Causal Assumption Testing”).

#### Model construction.

We constructed the causal model through an iterative process of hypothesis formulation, graphical representation, and empirical validation (see section “Model validation” and causal flow diagram, Fig H in S1 Appendix). The DAG was built using domain knowledge and experimental design constraints:

Randomised treatments (vaccine *V*, diet *D*, habitat *H*) have no parent nodes. Sex *S* enters as an exogenous pre-treatment biological attribute; balance of *S* across randomised arms is summarised in “Model validation”. We posited the habitat subgraph shown in Fig 3, with randomised *V* and exogenous *S* having no incoming directed edges from *H*; diet affects reproductive status *R*, body mass *M*, body fat *F*, parasite burden *P*, and vaccine response at endpoint *E*; and fat reserves *F* causally precede body mass *M*. Vaccine responsiveness *E* was modelled as affected by all other variables. Each variable includes an independent error term capturing unmeasured sources of variation.

Model construction was performed in Julia v1.12.6 [79] using the dagitty R package [87] via RCall.jl v0.14.13 [88] for causal identification and Turing.jl v0.44.5 [84] for Bayesian estimation. The DAG ${𝔾}_{VE}$ corresponding to ${\mathbb{C}}_{VE}$ was visualised using Tikz [89].

#### Model validation.

We validated ${\mathbb{C}}_{VE}$ in two parts [41,90]. **(i)** Under ${𝔾}_{VE}$, *D*, *H*, *V*, and *S* are exogenous, with no directed edges between them; paths between distinct members of {*D*,*H*,*V*,*S*} pass through colliders at descendants, so the DAG yields six pairwise marginal independencies ((D⫫S), (D⫫H), (D⫫V), (H⫫S), (H⫫V), and (S⫫V)) used as randomisation/balance checks. **(ii)** We fitted five mixed-model screens for (F⫫V∣D,H), (M⫫V∣D,H), (R⫫V∣D,H), (P⫫V∣D,H), and (P⫫V∣D,R,S,H) under the working graph with V→F, V→M, V→R, and V→P. These screens assess empirical compatibility with observed *V*–mediator alignment (weak *V* contributions after adjustment support internal coherence of the specification). Each check used generalised linear mixed models with appropriate link functions and random intercepts for mouse ID and immunisation protocol; support corresponded to *P* > 0.05 (two-sided). MixedModels.jl v5.1.0 [82] was used. All eleven checks met this criterion, consistent with retaining ${𝔾}_{VE}$ subject to substantive plausibility and the usual limits of *p*-value–based screening.

#### Model parameterisation, interventional simulation, and counterfactual prediction.

Bayesian models were fitted using the same prior specifications as the hierarchical models above, with the structural equations encoded as a system of regression models. All modelling was performed in Julia v1.12.6 [79] using Turing.jl v0.44.5 [84].

Following Pearl/Bareinboim, we distinguish *observational*, *interventional* (post-intervention under *do*(*P* = 0)), and *counterfactual* paired contrasts. We write $E_{factual}$ for the observational branch and $E_{do(P=0)}$ for posterior predictive *E* under *do*(*P* = 0) (interventional potential outcomes); contrasts $\Delta E=E_{do(P=0)}-E_{factual}$ instantiate the counterfactual layer. Julia code uses E_do_P0 for paired posterior draws of $E_{do(P=0)}$, with E_do_P0_mean and E_do_P0_sd for per-mouse summaries, and delta_E for ΔE.

#### Statistical identification.

The DAG ${𝔾}_{VE}$ was encoded as a Bayesian network representing each variable as a conditional distribution given its parents. Posterior distributions were estimated using Hamiltonian Monte Carlo with the No U-Turn Sampler [83], with 4 chains of 3,000 iterations each after 1,000 warmup iterations. Convergence diagnostics included $\hat{R}<1.01$, visual inspection of MCMC trace plots, and tabulated $\hat{R}$ with bulk and tail effective sample sizes for the observational (with parasites) and post-interventional (*do*(*P* = 0)) model fits (Table A in S1 Appendix).

#### Missing data imputation.

Twenty-two mice had missing fat scores (67 missing values total). We used Bayesian imputation within the structural model, where missing values were modelled as draws from their conditional distribution given observed parent variables: ${\dot{F}}_{missing}\sim \text{Normal}(\nu_{F},\sigma_{F})$, where $\nu_{F}$ and $\sigma_{F}$ were estimated from the data (see S1 Appendix, “Bayesian generative models for observational, interventional, and counterfactual inference” for details).

#### Interventional simulation and counterfactual estimation of anthelmintic treatment.

To evaluate potential benefits of anthelmintic treatment, we simulated the intervention *do*(*P* = 0) (complete parasite elimination) by structural substitution on the fitted SCM—equivalently, plug-in forward simulation under the mutilated graph [41]—rather than by an algebraic identification exercise using Pearl’s *do*-calculus on passive data alone. Prior predictive checks confirmed that both observational and post-interventional generative models were well-calibrated with appropriate prior coverage (Fig B in S1 Appendix). This involved fitting two Bayesian models: the observational model including all causal pathways, and the post-intervention model with the parasite effect on IgG1 OD at endpoint (*E*) set to zero while maintaining all other pathways (Fig 3C - 3D; see S1 Appendix, “Structural Causal Model Equations”).

For each mouse, we generated posterior predictive samples under both scenarios, calculating individual treatment effects as the difference between predictions under *do*(*P* = 0) and under the observational model. Effect sizes were calculated using Cohen’s *d* with pooled standard deviations, categorised as negligible (|*d*| < 0.2), small (0.2≤|d|<0.5), moderate (0.5≤|d|<0.8), or large (|d|≥0.8). This approach quantified both population-level improvements and individual heterogeneity in treatment benefits while propagating all sources of uncertainty through the causal model.

We calculated intervention effects using two complementary approaches: (1) **Population-level vaccination effect improvement**, which compares vaccination coefficients ($\beta_{V}$) between observational and post-interventional scenarios using mixed-effects models, quantifying how much larger the vaccination effect on diphtheria toxoid-specific IgG1 OD (*E*) becomes when parasites are eliminated; and (2) **Individual-level response improvement**, which calculates the percentage change in each mouse’s predicted IgG1 OD between observational (with parasites) and interventional (posterior predictive under *do*(*P* = 0)) scenarios. The population-level metric summarises shifts in $\beta_{V}$ on the IgG1 OD scale (a correlate, not clinical efficacy), whilst individual-level metrics capture the magnitude of benefit each animal would experience from parasite elimination on that same scale. (See additional details in S1 Appendix, “Effect Size Calculation and Clinical Significance”.)

While it would have been desirable to test interactions between sex and reproductive status, sample size limitations allowed only for testing the main effects of sex and reproductive status on vaccine responsiveness under the post-interventional (*do*(*P* = 0)) predictions (see S1 Appendix, “Bayesian generative models for observational, interventional, and counterfactual inference” for full details).

## Supporting information

S1 AppendixTable A MCMC diagnostics for the SCM intervention models.Convergence ($\hat{R}$) and effective sample sizes (bulk and tail ESS) for key parameters in the observational (with parasites) and post-interventional (*do*(*P* = 0)) generative models used to simulate anthelmintic intervention effects. Table B in S1 Appendix. Arm-level summary of IgG1 OD by immunisation history, habitat, and diet. Descriptive means and standard deviations of DTV-specific IgG1 optical density (OD) for A/D/AD/DA/DD arms stratified by habitat and diet (*n* = 222 observations; > 7 days after the most recent immunisation visit). Table C in S1 Appendix. Assay-floor observations by habitat and immunisation arm. Counts among antigen-containing arms (D, AD, DA, DD) for observations taken >7 days after the most recent immunisation visit. An observation was classified as assay-floor when blank-centred IgG1 OD was at or below the per-plate cut-off. Individual counts refer to unique mouse IDs with at least one qualifying bleed in that habitat–arm stratum. Fig A in S1 Appendix. Temporal dynamics analysis of vaccine response kinetics. A, Vaccine response trajectories by habitat from 311 observations (213 laboratory, 98 wild animals). Laboratory mice (blue, mean OD = 1.20) consistently achieve higher responses than wild mice (red, mean OD = 0.64), but both populations show similar temporal patterns. Individual data points show actual responses, whilst trend lines indicate population-level patterns. Shaded bands represent 95% confidence intervals around the LOESS smoothed trends. B, Distribution of individual peak response timing across all animals using temporal data. The median peak time is 22.0 days (IQR: 14.0–35.0 days), reflecting vaccination dynamics from the field study. This analysis demonstrates that habitat affects response magnitude more than timing, with laboratory animals showing 1.9-fold higher responses than wild animals. Fig B in S1 Appendix. Validation of prior predictive distributions for observational and post-interventional generative models. A, Prior predictive check for the observational model (with parasites). Histograms compare prior predictions (light blue) against observed standardised vaccine response data (red) with vertical dashed lines indicating respective means, confirming that the priors produce biologically plausible vaccine responses. B, Prior predictive check for the post-interventional generative model (without parasites). The histogram-based comparison shows prior predictions under the intervention *do*(*P* = 0) against observed data, validating the model’s ability to predict vaccine responses under *do*(*P* = 0) whilst maintaining appropriate coverage of the parameter space. Together, these panels demonstrate that both models are well-calibrated with weakly informative priors that provide sufficient regularisation for stable Bayesian inference whilst covering the observed data appropriately. Fig C in S1 Appendix. Data characteristics and model validation for vaccine responsiveness analysis. A, Distribution of standardised vaccine response measurements (DTV-specific IgG1 optical density, $\log_{10}$-transformed) across all experimental conditions. The histogram shows the range and distribution of vaccine responses observed in both laboratory and wild wood mice, demonstrating sufficient variation for causal inference whilst maintaining a roughly normal distribution suitable for linear modelling approaches. B, Relationship between parasite burden (continuous worm counts) and vaccine responsiveness (IgG1 OD), illustrating the negative association that motivated our structural causal model. C, As panel B, stratified by sex. Fig D in S1 Appendix. MCMC chains and posterior distributions of coefficients for Diet, Habitat, Time post immunisation, and vaccine formulations A, AD, D, DA, and DD. Fig E in S1 Appendix. MCMC convergence diagnostics for key structural causal models. A, Posterior distributions from the parasite burden effect model (P → E), showing parameter estimates for the direct causal effect of parasite infection on vaccine responsiveness. The plot displays both MCMC trace plots (left) and posterior density plots (right) for all model parameters, with multiple chains (different colours) demonstrating good mixing and convergence. B, Posterior distributions from the habitat effect model (H → E), showing parameter estimates for the total causal effect of wild habitat on vaccine responsiveness. Both models show well-behaved MCMC chains with $\hat{R}<1.01$ for all parameters, confirming reliable parameter estimation for the key causal inferences in our structural causal model. Fig F in S1 Appendix. Parasite count data analysis demonstrating need for zero-inflated modelling. A, Distribution of total parasite counts from 448 observations showing zero-inflation patterns. Cestodes show the most extreme zero-inflation (98.7% zeros), followed by pinworms (97.1%), *H. polygyrus* (95.5%), and fleas (96.9%). The long right tail and preponderance of zeros indicate that standard Poisson regression would be inappropriate. B, Zero-inflation assessment comparing uninfected versus infected animals using parasite burden data. The high proportion of uninfected animals reflects the ecological reality that many individuals are never exposed to parasites in natural populations. C, Distribution of non-zero parasite counts among infected animals, showing overdispersion patterns. *H. polygyrus* shows the highest variance/mean ratio (86.3), followed by pinworms (76.4) and cestodes (69.9), necessitating negative binomial rather than Poisson modelling. Together, these data characteristics strongly support the use of Zero-Inflated Negative Binomial (ZINB) models for realistic parasite effect estimation. Fig G in S1 Appendix. Comprehensive sensitivity analysis for model robustness assessment. A, E-values for unmeasured confounding sensitivity from manuscript analysis, showing the minimum strength of association an unmeasured confounder would need with both treatment and outcome to explain away observed effects. Point estimates (blue bars) show moderate E-values (habitat effect = 2.8, parasite effect = 1.9, sex effect = 3.0, age effect = 2.1), whilst confidence interval bounds (red bars) show E-values of 1.0 when intervals include the null, indicating limited robustness to unmeasured confounding. The dashed line at E-value = 2.0 represents the conventional robustness threshold. B, Prior sensitivity analysis for the habitat effect across three prior specifications (Conservative, Standard, Wide), showing coefficient of variation = 0.14, which exceeds the 0.1 threshold for robustness. Despite this sensitivity, all specifications yield negative estimates (−0.52, −0.58, −0.61), supporting the consistent finding that wild habitat reduces vaccine responsiveness. Fig H in S1 Appendix. Flow diagram of the SCM. The Structural Causal Model (SCM) process explicitly represents scientific hypotheses (1) as a directed acyclic graph (DAG). This DAG is mathematically encoded as a set of nested equations that describe the flow of causation between variables, and helps inform lab and field experimental design (3) and data collection (4). After processing, the data are used to test the validity of the causal assumptions underlying the SCM, e.g., marginal balance relations implied among exogenous nodes plus auxiliary mixed-model screens (main text “Model validation”), (5). If the assumptions are not supported, refinements of the SCM (6) are necessary. When all conditions are met, the SCM is identifiable and statistical models can be formulated to adjust for confounding (7). Parameters from these models can then be used as estimates of direct and indirect causal effects (8).(PDF)
