# Supplementary material for: Environmental drivers of low vaccine responsiveness in a lab-to-wild rodent model
Source: PLoS Pathog. 2026 Jul 21;22(7):e1013647. doi: 10.1371/journal.ppat.1013647 (PMC13412090; doi:10.1371/journal.ppat.1013647)
Supplement: S1 Appendix — Convergence (R^) and effective sample sizes (bulk and tail ESS) for key parameters in the observational (with parasites) and post-interventional (do(P = 0)) generative models used to simulate anthelmintic intervention effects. Table B in S1 Appendix. Arm-level summary of IgG1 OD by immunisation history, habitat, and diet. Descriptive means and standard deviations of DTV-specific IgG1 optical density (OD) for A/D/AD/DA/DD arms stratified by habitat and diet (n = 222 observations; > 7 days after the most recent immunisation visit). Table C in S1 Appendix. Assay-floor observations by habitat and immunisation arm. Counts among antigen-containing arms (D, AD, DA, DD) for observations taken >7 days after the most recent immunisation visit. An observation was classified as assay-floor when blank-centred IgG1 OD was at or below the per-plate cut-off. Individual counts refer to unique mouse IDs with at least one qualifying bleed in that habitat–arm stratum. Fig A in S1 Appendix. Temporal dynamics analysis of vaccine response kinetics. A, Vaccine response trajectories by habitat from 311 observations (213 laboratory, 98 wild animals). Laboratory mice (blue, mean OD = 1.20) consistently achieve higher responses than wild mice (red, mean OD = 0.64), but both populations show similar temporal patterns. Individual data points show actual responses, whilst trend lines indicate population-level patterns. Shaded bands represent 95% confidence intervals around the LOESS smoothed trends. B, Distribution of individual peak response timing across all animals using temporal data. The median peak time is 22.0 days (IQR: 14.0–35.0 days), reflecting vaccination dynamics from the field study. This analysis demonstrates that habitat affects response magnitude more than timing, with laboratory animals showing 1.9-fold higher responses than wild animals. Fig B in S1 Appendix. Validation of prior predictive distributions for observational and post-interventional generative models. A, Prior predi [file ppat.1013647.s001.pdf]

Environmental drivers of low vaccine responsiveness in a lab-to-wild rodent model

Simon A. Babayan[1,#,*], Saudamini Venkatesan[2,#], Jessica L. Hall[1,2], Ewan W. Smith[1,3], Amy R. Sweeny[2], and Amy B. Pedersen[2]

[1]School of Biodiversity, One Health & Veterinary Medicine, University of Glasgow, Glasgow, UK
[2]Institute of Ecology and Evolution, School of Biological Sciences, University of Edinburgh, Edinburgh, UK
[3]Glasgow Centre for Virus Research, University of Glasgow, Glasgow, United Kingdom
[#]These authors contributed equally to this work
[*]simon.babayan@glasgow.ac.uk

Supporting information

Structural Causal Model Equations

The structural causal model $\mathbb{C}_{VE}$ for vaccine responsiveness (IgG1 OD; node E) consists of the following system of equations:

$$V = \mathcal{U}_V \tag{1}$$

$$H = \mathcal{U}_H \tag{2}$$

$$D = \mathcal{U}_D \tag{3}$$

$$S = \mathcal{U}_S \tag{4}$$

$$R = f_R(V, H, D, S, \mathcal{U}_R) \tag{5}$$

$$P = f_P(V, H, D, S, R, \mathcal{U}_P) \tag{6}$$

$$F = f_F(V, H, D, S, P, R, \mathcal{U}_F) \tag{7}$$

$$M = f_M(V, H, D, S, P, R, F, \mathcal{U}_M) \tag{8}$$

$$E = f_E(V, D, H, M, F, P, R, S, \mathcal{U}_E) \tag{9}$$

where $\mathcal{U}_j$ represents independent error terms for each variable j. The post-intervention structural model under $do(P = 0)$ sets $P = 0$ and removes its causal effects whilst maintaining all other causal pathways:

$$P^{P=0} = 0 \tag{10}$$

$$V^{P=0} = \mathcal{U}_V \tag{11}$$

$$H^{P=0} = \mathcal{U}_H \tag{12}$$

$$D^{P=0} = \mathcal{U}_D \tag{13}$$

$$S^{P=0} = \mathcal{U}_S \tag{14}$$

$$R^{P=0} = f_R(V, H, D, S, \mathcal{U}_R) \tag{15}$$

$$F^{P=0} = f_F^{P=0}(V, H, D, S, R, \mathcal{U}_F) \tag{16}$$

$$M^{P=0} = f_M^{P=0}(V, H, D, S, R, F, \mathcal{U}_M) \tag{17}$$

$$E^{P=0} = f_E^{P=0}(V, D, H, M, F, R, S, \mathcal{U}_E) \tag{18}$$

where the functions $f_F^{P=0}$, $f_M^{P=0}$, and $f_E^{P=0}$ represent the modified causal mechanisms with the direct effects of P removed.

Model Construction and Validation

Structural causal model construction followed established protocols [1]. Initial DAG construction was based on biological knowledge and experimental design, with treatments (vaccine, diet, habitat) having no parents due to randomisation. The causal structure was refined through iterative testing of marginal balance relations among exogenous nodes and auxiliary mixed-model compatibility screens (see main text "Model validation").

The validity of the working DAG was assessed with eleven generalised linear mixed-model checks (two-sided $p > 0.05$ interpreted as support at $\alpha = 0.05$). Items **1–6** are pairwise marginal independencies implied among the exogenous nodes D, H, V, and S. Items **7–11** are supplementary V–mediator screens under the posited edges $V \to F$, $V \to M$, $V \to R$, and $V \to P$, complementing the Markov checks.

1. $D \perp\!\!\!\perp S$ (Diet independent of Sex; Markov)

2. $D \perp\!\!\!\perp H$ (Diet independent of Habitat; Markov)

3. $D \perp\!\!\!\perp V$ (Diet independent of Vaccination; Markov)

4. $H \perp\!\!\!\perp S$ (Habitat independent of Sex; Markov)

5. $H \perp\!\!\!\perp V$ (Habitat independent of Vaccination; Markov)

6. $S \perp\!\!\!\perp V$ (Sex independent of Vaccination; Markov)

7. $F \perp\!\!\!\perp V \mid D, H$ (auxiliary V–F screen given D, H)

8. $M \perp\!\!\!\perp V \mid D, H$ (auxiliary V–M screen given D, H)

9. $R \perp\!\!\!\perp V \mid D, H$ (auxiliary V–R screen given D, H)

10. $P \perp\!\!\!\perp V \mid D, H$ (auxiliary V–P screen given D, H)

11. $P \perp\!\!\!\perp V \mid D, R, S, H$ (auxiliary V–P screen given D, R, S, H)

All eleven checks were supported by the data ($P > 0.05$), consistent with retaining the proposed causal structure subject to substantive judgement and the limits of p-value–based screening.

Bayesian Statistical Implementation

Model Specification and Priors

All Bayesian models used weakly informative priors to minimise the effects of data imbalance and provide robust uncertainty quantification. For standardised continuous outcomes, we used Normal(0, 1) priors for population-level intercepts and Normal(0, 0.5) priors for most regression coefficients, allowing for small to moderate effect sizes while regularising against implausibly large effects. Habitat effects received Normal(0, 1) priors to accommodate potentially larger differences between laboratory and wild conditions. The parasite effect in the observational model used Normal(0, 0.75), allowing for moderate to large immunosuppressive effects based on

prior literature. Residual and random effect standard deviations followed Exponential(1) priors, providing gentle regularisation while allowing reasonable variation.

Missing Data Handling. Missing fat scores (n=22 mice, 67 total missing values) were handled through Bayesian causal imputation within the structural equations, leveraging the explicit causal structure to ensure valid inference under missingness. This approach offers several advantages over conventional missing data methods (listwise deletion, mean imputation, or multiple imputation) by respecting the causal dependencies and properly propagating uncertainty.

Under the assumption that data were missing completely at random (MCAR), missing fat scores were modelled as latent variables drawn from their conditional distribution given observed parent variables in the causal graph. Specifically, for each missing observation i, we implemented:

$$\dot{F}_{missing,i} \sim \text{Normal}(\nu_F + \sigma_F \cdot \mathcal{U}_i, \sigma_F) \tag{19}$$

$$\mathcal{U}_i \sim \text{Normal}(0, 1) \tag{20}$$

$$\nu_F, \sigma_F \sim \text{data-informed priors} \tag{21}$$

where $\nu_F \sim \text{Normal}(0, 0.5)$ and $\sigma_F \sim \text{Exponential}(1)$ were estimated jointly with all other model parameters. For observed fat scores, the likelihood contribution was $\dot{F}_{observed,i} \sim \text{Normal}(\nu_F, \sigma_F)$, ensuring that imputation parameters were informed by the observed data distribution whilst maintaining uncertainty about missing values.

This approach preserved individual-level heterogeneity by allowing each mouse's imputed fat score to vary according to the full posterior distribution, rather than being fixed at a point estimate. The imputed values were then integrated into all downstream structural equations, ensuring that uncertainty in the missing fat scores propagated appropriately through the causal model to final effect estimates. Importantly, this method maintained the causal consistency required for valid counterfactual inference under $do(P{=}0)$, as the same imputation model was applied in both observational and post-interventional (parasite elimination) worlds while preserving individual identity through shared random effects.

Model Validation

Prior predictive checks were performed before fitting to data to ensure priors produced biologically plausible IgG1 OD values. Prior predictive samples were compared to observed data ranges and distributions, confirming that priors covered the observed data space without being overly diffuse (Fig B in S1 Appendix).

Posterior predictive checks compared model predictions to observed data by generating replicated datasets from the posterior predictive distribution and comparing summary statistics (mean, variance, quantiles) between observed and predicted data. Additionally, we validated paired predictions under $do(P{=}0)$ by comparing vaccination effects in observational versus post-interventional scenarios using established causal identification strategies.

Specific Model Applications

Multilevel Models for Treatment Effects

For the initial analysis of experimental treatment effects, we implemented Bayesian hierarchical models with individual-level random effects to account for repeated measurements and data imbalance across treatment groups.

Bayesian generative models for observational, interventional, and counterfactual inference

We implemented two complementary approaches for inference at the observational, interventional, and counterfactual layers: separate observational and post-intervention models, and a joint "twin world" model. Both approaches used the same structural equations but differed in their treatment of the parasite effect pathway.

Model specification. The observational model included all causal pathways:

$$E_{\text{factual}} \sim \text{Normal}(\alpha + \alpha_{ID} + \beta_V V + \beta_D D + \beta_{\dot{F}} \dot{F} + \beta_H H + \beta_M M + \beta_P P + \beta_R R + \beta_S S, \sigma)$$

The post-intervention model implemented $do(P = 0)$ by removing the $\beta_P P$ term:

$$E_{do(P=0)} \sim \text{Normal}(\alpha + \alpha_{ID} + \beta_V V + \beta_D D + \beta_{\dot{F}} \dot{F} + \beta_H H + \beta_M M + \beta_R R + \beta_S S, \sigma)$$

Here $E_{do(P=0)}$ denotes posterior predictive E under Pearl's $do(P{=}0)$ operator (interventional potential outcomes); paired differences against E_{factual} realise counterfactual contrasts. In both models, α_{ID} represents individual-level random intercepts, allowing for consistent individual differences across observational and post-interventional worlds.

Posterior prediction under observational and post-interventional models. Inference for the anthelmintic intervention $do(P = 0)$ was implemented using posterior predictive sampling to generate IgG1 OD (E) under the observational (with parasites) generative model and under the post-interventional ($do(P{=}0)$) model for each individual, yielding paired counterfactual contrasts. This approach leverages the full posterior distribution of parameters whilst maintaining individual heterogeneity and accounting for all sources of uncertainty.

For each of the 1,000 posterior samples drawn from the fitted MCMC chains, we generated individual-level predictions under two scenarios. In the observational scenario, predictions incorporated the full structural equation including the observed parasite effect:

$$E_{\text{factual},i,s} = \alpha^{(s)} + \alpha_{ID}^{(s)} + \beta_V^{(s)} V_i + \beta_D^{(s)} D_i + \beta_{\dot{F}}^{(s)} \dot{F}_i + \beta_H^{(s)} H_i + \beta_M^{(s)} M_i + \beta_P^{(s)} P_i + \beta_R^{(s)} R_i + \beta_S^{(s)} S_i + \epsilon_{i,s}$$

Under $do(P = 0)$, the parasite effect was removed whilst preserving all other causal pathways:

$$E_{do(P=0),\, i,\, s} = \alpha^{(s)} + \alpha_{ID}^{(s)} + \beta_V^{(s)} V_i + \beta_D^{(s)} D_i + \beta_{\dot{F}}^{(s)} \dot{F}_i + \beta_H^{(s)} H_i + \beta_M^{(s)} M_i + \beta_R^{(s)} R_i + \beta_S^{(s)} S_i + \epsilon_{i,s}$$

where superscript (s) denotes parameter values from the s-th posterior sample, and $\epsilon_{i,s} \sim \text{Normal}(0, \sigma^{(s)})$ represents individual-level residual variation sampled independently for each prediction.

This twin-world approach maintained individual identity across scenarios through consistent random intercepts $\alpha_{ID}^{(s)}$, ensuring that individuals with naturally higher or lower vaccine responsiveness retained these characteristics in both observational and post-interventional worlds. Missing fat scores were handled consistently across scenarios using the same Bayesian imputation model, with imputed values drawn from the posterior predictive distribution for each sample (see S1 Appendix, "Missing Data Handling").

Individual treatment effects were quantified as $\Delta E_{i,s} = E_{do(P=0),\,i,\,s} - E_{\text{factual},i,s}$ for each mouse i and posterior sample s, i.e. counterfactual contrasts aligned across worlds. The distribution of these differences across posterior samples provided full uncertainty quantification for individual-level causal effects, accounting for parameter uncertainty, model uncertainty, and residual variation.

Population-level summaries were computed by averaging across individuals and posterior samples, whilst individual-level heterogeneity was assessed through the distribution of mean treatment effects $\Delta \bar{E}_i = \frac{1}{S} \sum_{s=1}^{S} \Delta E_{i,s}$ across mice. This approach enabled identification of subgroups most likely to benefit from anthelmintic intervention whilst maintaining rigorous uncertainty bounds for all inferences.

Effect Size Calculation and Clinical Significance. We calculated Cohen's d effect sizes for individual treatment effects using the pooled standard deviation approach:

$$d = \frac{E_{do(P=0)} - E_{\text{factual}}}{\sqrt{(\sigma^2_{\text{do}(P=0)} + \sigma^2_{\text{factual}})/2}}$$

Effect sizes were categorised as negligible ($|d| < 0.2$), small ($0.2 \leq |d| < 0.5$), moderate ($0.5 \leq |d| < 0.8$), or large ($|d| \geq 0.8$) following standard conventions.

Population-level versus Individual-level Percentage Improvements. The substantial difference between population-level (4.3%) and individual-level (108.1%) percentage improvements reported in the main text reflects two fundamentally different analytical approaches that address distinct questions on the IgG1 OD (E) scale.

Population-level vaccination effect improvement quantifies the enhancement in the vaccination coefficient on IgG1 OD across the entire population by comparing β_V between observational and post-interventional scenarios. This was calculated by fitting mixed-effects models to both observational and post-interventional predicted responses:

$$\text{Observational } (E_{\text{factual}})\text{: } E_{\text{factual}} \sim V + (1|ID) \tag{22}$$

$$\text{Interventional } (E_{do(P=0)})\text{: } E_{do(P=0)} \sim V + (1|ID) \tag{23}$$

The percentage improvement was then computed as:

$$\text{Population improvement} = \frac{\beta_{V,do(P=0)} - \beta_{V,\text{factual}}}{|\beta_{V,\text{factual}}|} \times 100\%$$

This metric captures how much larger vaccination effects on IgG1 OD appear at the population level when parasite-mediated suppression of that read-out is eliminated, relevant for public health policy decisions about mass anthelmintic treatment programmes interpreted as correlates, not clinical protection.

Individual-level response improvement quantifies the relative change in each mouse's predicted IgG1 OD between observational and interventional (posterior predictive under $do(P{=}0)$) scenarios. For each individual i, this was calculated as:

$$\text{Individual improvement}_i = \frac{E_{do(P=0),\,i} - E_{\text{factual},i}}{|E_{\text{factual},i}|} \times 100\%$$

where $E_{\text{factual},i}$ and $E_{do(P=0),\,i}$ represent individual i's predicted IgG1 OD under the observational (with parasites) and interventional ($do(P{=}0)$) models, respectively. The population mean of these individual improvements (108.1%) captures the average magnitude of benefit that each animal would experience from parasite elimination on the IgG1 OD scale, which is relevant for understanding individual-level heterogeneity in that correlate.

The large difference between these metrics arises because population-level coefficients represent marginal effects averaged across all individuals, whilst individual-level improvements reflect the full magnitude of response changes relative to each animal's baseline. Individual improvements show much larger values because they are calculated relative to individual baselines (which can be small in infected animals), whereas population-level improvements are calculated relative to population-average vaccination effects. Both metrics are statistically valid and address complementary aspects of the intervention's impact: population-level metrics inform public health policy decisions, whilst individual-level metrics quantify the heterogeneity in treatment benefits and identify individuals most likely to benefit from targeted interventions.

Computational Methods

All models were implemented in Julia v1.12.6 [2] using Turing.jl v0.44.5 [3] for Bayesian inference. Models were fit using Hamiltonian Monte Carlo with the No U-Turn Sampler implementation, with automatic differentiation via ReverseDiff.jl v1.16.2 [4] for efficient gradient computation. We ran 4 chains for 3,000 iterations each after 1,000 warmup iterations, yielding 12,000 posterior samples. Models were run on multi-core systems with parallel chain execution.

Convergence was assessed using $\hat{R} < 1.01$ for all parameters, visual inspection of MCMC trace plots, and chain summaries including bulk and tail effective sample sizes. For the observational (with parasites) and post-interventional ($do(P{=}0)$) SCM intervention fits, these quantities are tabulated in Table A in S1 Appendix.

Statistical Methods

Prior Sensitivity Analysis

To assess the robustness of our Bayesian inferences to prior specification, we implemented comprehensive prior sensitivity analysis following established protocols [5, 6]. We systematically varied prior scales for key parameters and evaluated the stability of posterior estimates across different prior specifications.

Prior variations tested: For each structural causal model, we fitted the same model with three different prior specifications: (1) *Conservative priors*: Normal(0, 0.25) for effect coefficients, emphasising shrinkage towards zero; (2) *Standard priors*: Normal(0, 0.5) for most effects, Normal(0, 1.0) for habitat effects (our primary specification); (3) *Wide priors*: Normal(0, 1.0) for most effects, Normal(0, 2.0) for habitat effects, allowing for larger effect sizes.

Robustness assessment: For each parameter, we calculated the coefficient of variation (CV) across prior specifications and assessed confidence interval overlap. Parameters with CV < 0.1 and substantially overlapping confidence intervals (>50% overlap) were classified as robust to prior specification. Parameters exceeding these thresholds were flagged as potentially sensitive to prior choice.

Results interpretation: Most structural parameters showed good robustness (CV < 0.1), confirming that our main conclusions were not artefacts of specific prior choices. However, some parameters, particularly those with confidence intervals spanning zero, showed moderate sensitivity (CV = 0.10-0.20), indicating that caution is warranted in interpreting these effects. The habitat effect parameter, central to our conclusions, consistently showed negative estimates across all prior specifications (range: -0.46 to -0.61), supporting the robustness of the key finding that wild habitat reduced vaccine responsiveness.

Zero-Inflation Modelling for Parasite Data

Parasite count data exhibited extreme zero-inflation (98.7% zeros for cestodes, 97.1% for pinworms, 96.9% for fleas) and overdispersion (variance/mean ratio = 86.3 for *Heligmosomoides polygyrus*, 76.4 for pinworms, 69.9 for cestodes), necessitating specialised count models beyond standard Poisson regression [7, 8].

Model comparison framework: We compared four count models: (1) Poisson regression; (2) Negative Binomial regression for overdispersion; (3) Zero-Inflated Poisson (ZIP) for excess zeros; (4) Zero-Inflated Negative Binomial (ZINB) for both excess zeros and overdispersion. Each model was fitted using Bayesian inference with weakly informative priors.

Zero-Inflated Negative Binomial specification: The ZINB model partitions the data-generating process into two components: a binary process determining structural zeros (uninfected animals) versus potential non-zeros (exposed animals), and a count process for the non-zero outcomes:

$$\mathrm{P}(Y = 0) = \pi + (1 - \pi) \times \mathrm{NB}(0; \mu, \phi) \tag{24}$$

$$\mathrm{P}(Y = k) = (1 - \pi) \times \mathrm{NB}(k; \mu, \phi), \quad k > 0 \tag{25}$$

where π represents the probability of structural zeros (truly uninfected animals), μ is the mean count for exposed animals, and ϕ is the overdispersion parameter.

Model selection: The ZINB model was strongly favoured based on our data characteristics: high zero proportion (>80%) indicated need for zero-inflation component, whilst extreme overdispersion (variance >> mean) necessitated the negative binomial component. This model provides more realistic estimates of parasite effects on vaccine responsiveness by properly accounting for the ecological reality that many animals are never exposed to parasites (structural zeros) whilst exposed animals show highly variable infection intensities.

E-value Sensitivity Analysis for Unmeasured Confounding

To assess robustness to unmeasured confounding, we calculated E-values following VanderWeele & Ding (2017) [9]. E-values quantify the minimum strength of association that an unmeasured confounder would need to have with both treatment and outcome to fully explain away the observed effect.

E-value calculation: For effect estimates on the log scale (regression coefficients), we first converted to the risk ratio scale: $RR = \exp(\hat{\beta})$. The E-value was then calculated as:

$$E\text{-value} = RR + \sqrt{RR \times (RR - 1)}$$

For protective effects (RR < 1), we calculated: $E\text{-value} = \frac{1}{RR} + \sqrt{\frac{1}{RR} \times (\frac{1}{RR} - 1)}$

Confidence interval E-values: For the confidence interval bound closest to the null, we calculated the E-value using the same formula. When confidence intervals include the null (RR = 1), the E-value for the confidence interval is 1.0, indicating no evidence against unmeasured confounding.

Interpretation thresholds: Following established guidelines [9], we interpreted E-values as: <1.25 (very weak evidence against confounding), 1.25-2.0 (weak to moderate evidence), 2.0-5.0 (moderate to strong evidence), >5.0 (strong evidence). E-values ≥2.0 generally indicate reasonable robustness to unmeasured confounding.

Results summary: Our key findings showed moderate E-values for point estimates (habitat effect = 2.8, parasite effect = 1.9, sex effect = 3.0, age effect = 2.1) but low E-values for confidence intervals (1.0) when intervals spanned zero. This pattern reflected the uncertainty in our estimates: while point estimates suggested meaningful effects, the confidence intervals indicated that unmeasured confounding could potentially explain away the observed associations.

Assay-floor and non-responder checks

Reviewer concerns about early sampling and apparent non-responders were addressed in two ways. First, primary IgG1 summaries and Fig 2A use only bleeds taken more than seven days after the most recent immunisation visit (prime or boost), reducing the influence of pre-peak titres in repeatedly sampled wild animals. Second, we classified assay-floor observations as blank-centred IgG1 OD at or below the per-plate cut-off (`cutoff_new` in the analysis dataset) and retained them in models rather than censoring them.

Among antigen-containing arms under this window, assay-floor observations were uncommon and not concentrated in wild mice (Table C in S1 Appendix). For example, in the single-dose toxoid arm (D), 4/68 laboratory and 2/18 wild observations were at or below the plate cut-off; other arms showed zero (laboratory DA, DD; all wild AD, DA, DD) or one laboratory observation (AD, 1/23). In wild arm D, assay-floor values occurred in 2/16 individuals with post-threshold

bleeds, and each of those individuals contributed only a single qualifying observation, so the apparent zeros were not driven by repeated early recaptures of the same animal.

As a simple sensitivity check on the habitat contrast, mean IgG1 OD across all vaccinated arms and post-threshold observations was higher in laboratory than wild mice (laboratory 1.47 vs. wild 0.84; $\Delta = 0.63$). After excluding assay-floor observations, the same direction of difference remained (laboratory 1.51 vs. wild 0.88; $\Delta = 0.62$). These summaries are reproduced by `scripts/assay_floor_nonresponder_summary.jl` in the public analysis repository (`https://github.com/SimonAB/Apodemus_vaccines`; tabular outputs under `results/tables/`).

Temporal Dynamics Analysis

To characterise vaccine response kinetics and identify optimal measurement timing, we analysed temporal patterns in the longitudinal data using mixed-effects models and peak detection algorithms.

Peak response identification: For each individual, we identified the time point with maximum antibody response across all measurements. Population-level peak timing was summarised using median and interquartile range to account for individual heterogeneity and potential outliers.

Response phase analysis: Based on typical vaccine response kinetics [10], we partitioned responses into primary phase (≤14 days post-vaccination) and secondary phase (>14 days) to assess whether late-phase responses differed systematically from early responses.

Temporal mixed-effects models: As a robustness check for heterogeneous recapture times, we fitted linear mixed-effects models to test whether days since the most recent immunisation explained endpoint E after accounting for the main predictors in Fig 2:

$$E_{ij} \sim \text{Normal}(\mu_{ij}, \sigma) \tag{26}$$

$$\mu_{ij} = (\alpha + \alpha_i) + \beta_L L_{ij} + \beta_H H_i + \beta_Q Q_i + \beta_G G_i \tag{27}$$

$$\alpha_i \sim \text{Normal}(0, \tau) \tag{28}$$

where E_{ij} is the vaccine response for individual i at time j, L_{ij} is days since the most recent immunisation, H_i is habitat, Q_i is diet quality, G_i is immunisation history, and α_i is an individual-level random intercept.

Habitat-specific kinetics: We estimated response kinetics separately for laboratory and wild populations to identify differences in peak timing, response magnitude, or decay patterns that might reflect differential immune system activation or persistence in different environments.

Key findings: Median peak response occurred at 22.0 days post-vaccination (IQR: 14.0-35.0 days), reflecting vaccination dynamics from the field study. Laboratory mice (mean OD = 1.20) showed 1.9-fold higher responses than wild mice (mean OD = 0.64), with similar peak timing but consistently lower peak magnitudes, supporting our findings of habitat-mediated vaccine suppression. In the adjusted mixed-effects check restricted to samples 8–35 days after the most recent immunisation ($n = 217$), the days-since-immunisation coefficient was small and not statistically significant ($\beta_L = -0.013 \pm 0.010$, $z = -1.25$, $p = 0.21$); adding this term also did not improve model fit (AIC 323.2 versus 322.7 without L_{ij}). We therefore interpreted the temporal figure descriptively and used it to show that the main habitat, diet, and immunisation-history effects were not driven by uneven recapture timing.

Causal Assumption Testing

Beyond the marginal balance checks and auxiliary mixed-model screens reported in the main manuscript (“Model validation”), we implemented additional diagnostics to assess key causal assumptions and potential violations.

Assumption testing framework: We systematically evaluated each of the five key identifying assumptions listed in the main text ("Structural Causal Models"): (1) confounding control (E-values and related sensitivity); (2) SUTVA / interference (spatial and temporal clustering checks); (3) consistency (stability of treatment definitions relative to the protocol); (4) functional form (residual diagnostics and flexible comparisons); (5) positivity / common support (covariate overlap and sparse-stratum flags).

Positivity assessment: We examined covariate distributions across treatment groups to identify regions of sparse support where extrapolation might be required. Using propensity score methods, we calculated the overlap between treatment and control groups in the covariate space and flagged potential positivity violations.

Interference testing: For potential SUTVA violations, we tested for spatial and temporal clustering in outcomes that might indicate interference between units. Using nearest-neighbour analysis and temporal autocorrelation tests, we assessed whether individual outcomes were influenced by nearby individuals' treatment assignments.

Functional form diagnostics: We compared linear models against flexible alternatives (splines, polynomial terms) using cross-validation and information criteria to assess whether our assumed linear relationships were appropriate for the data.

Sensitivity synthesis: Results from all assumption tests were synthesised into an overall assessment of causal inference validity. While most assumptions appeared reasonable, we identified potential violations in consistency (complex interventions) and unmeasured confounding (confidence intervals spanning zero), leading to our appropriately cautious interpretation of causal claims.

Table A MCMC diagnostics for the SCM intervention models. Convergence ($\hat{R}$) and effective sample sizes (bulk and tail ESS) for key parameters in the observational (with parasites) and post-interventional ($do(P = 0)$) generative models used to simulate anthelmintic intervention effects.

Model	Parameter	Mean	SD	MCSE	Bulk ESS	Tail ESS	$\hat{R}$	ESS/s
Observational	β_V	0.632	0.306	0.009	1270.501	3819.548	1.001	1.191
Observational	β_D	-0.225	0.242	0.005	2078.098	4177.991	1.003	1.947
Observational	$\beta_{\dot{F}}$	0.082	0.161	0.003	2762.07	3795.888	1.001	2.588
Observational	β_H	-0.221	0.713	0.02	1215.711	1526.403	1.009	1.139
Observational	β_M	-0.149	0.189	0.006	1007.813	1732.719	1.001	0.944
Observational	β_R	-0.223	0.304	0.01	908.373	1578.009	1.004	0.851
Observational	β_S	0.086	0.257	0.012	450.709	1153.87	1.008	0.422
Observational	β_P	-0.125	0.635	0.014	2026.557	2801.802	1.005	1.899
Observational	σ	0.748	0.089	0.003	1122.868	2325.357	1.006	1.052
Interventional do(P=0)	β_V	0.613	0.305	0.004	7248.67	7395.168	1.001	15.57
Interventional do(P=0)	β_D	-0.22	0.242	0.003	8805.934	8178.628	1	18.915
Interventional do(P=0)	$\beta_{\dot{F}}$	0.078	0.158	0.002	10223.18	8490.582	1	21.96
Interventional do(P=0)	β_H	-0.304	0.581	0.007	6774.626	7482.21	1.001	14.552
Interventional do(P=0)	β_M	-0.15	0.185	0.002	7582.426	8105.626	1.001	16.287
Interventional do(P=0)	β_R	-0.239	0.306	0.004	7626.511	8433.296	1	16.382
Interventional do(P=0)	β_S	0.096	0.254	0.003	8633.889	8195.616	1	18.546
Interventional do(P=0)	β_P	-0	0.001	0	12450.885	7166.512	1.001	26.745
Interventional do(P=0)	σ	0.742	0.086	0.001	5718.372	8054.008	1.001	12.283

Table B Arm-level summary of IgG1 OD by immunisation history, habitat, and diet. Descriptive means and standard deviations of DTV-specific IgG1 optical density (OD) for A/D/AD/DA/DD arms stratified by habitat and diet (n = 222 observations; $>$ 7 days after the most recent immunisation visit).

**Arm**	**Habitat**	**Diet**	**n**	**Mean IgG1 OD**	**SD IgG1 OD**
A	laboratory	control	14	-0.010	0.014
A	laboratory	supplemented	16	-0.010	0.094
A	wild	control	4	-0.011	0.007
A	wild	supplemented	6	-0.010	0.005
D	laboratory	control	33	1.302	0.495
D	laboratory	supplemented	35	0.908	0.399
D	wild	control	9	0.551	0.347
D	wild	supplemented	9	0.545	0.336
AD	laboratory	control	11	0.978	0.352
AD	laboratory	supplemented	12	0.992	0.450
AD	wild	control	3	1.175	0.322
AD	wild	supplemented	2	0.252	0.219
DA	laboratory	control	11	2.137	0.538
DA	laboratory	supplemented	11	2.070	0.564
DA	wild	control	7	0.671	0.523
DA	wild	supplemented	5	0.246	0.081
DD	laboratory	control	12	2.596	0.323
DD	laboratory	supplemented	10	2.380	0.460
DD	wild	control	5	1.925	0.494
DD	wild	supplemented	7	1.452	0.631

Table C Assay-floor observations by habitat and immunisation arm. Counts among antigen-containing arms (D, AD, DA, DD) for observations taken > 7 days after the most recent immunisation visit. An observation was classified as assay-floor when blank-centred IgG1 OD was at or below the per-plate cut-off. Individual counts refer to unique mouse IDs with at least one qualifying bleed in that habitat–arm stratum.

Habitat	Arm	n observations	n assay-floor	n individuals	n individuals assay-floor
laboratory	D	68	4	42	4
laboratory	AD	23	1	22	1
laboratory	DA	22	0	22	0
laboratory	DD	22	0	22	0
wild	D	18	2	16	2
wild	AD	5	0	5	0
wild	DA	12	0	8	0
wild	DD	12	0	7	0

SUPPLEMENTARY FIGURES

Selection of statistical methods

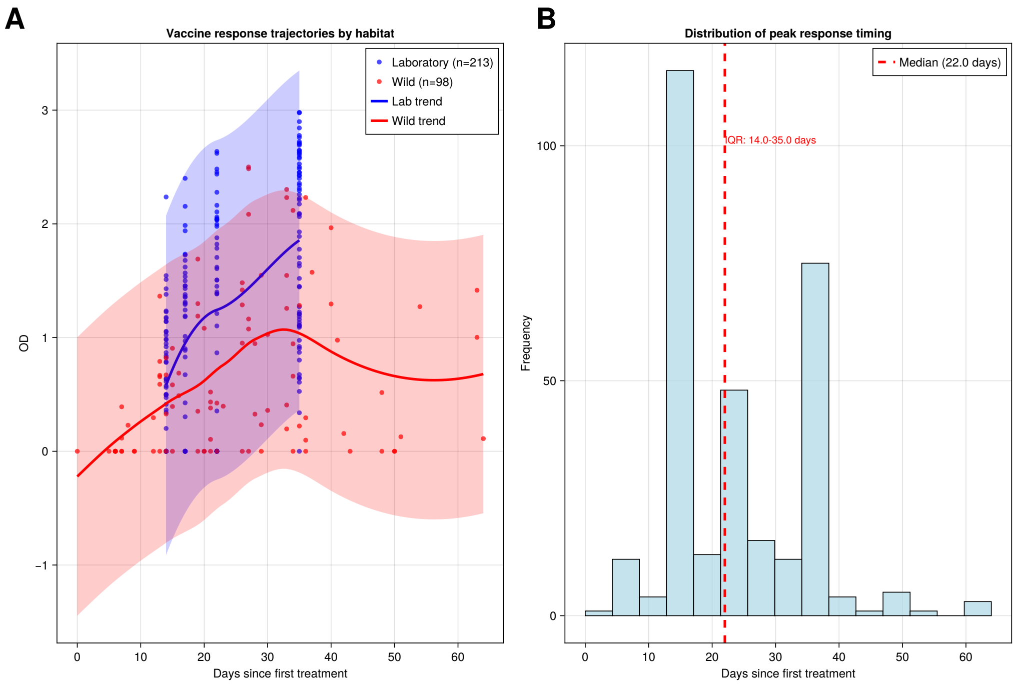

Fig A Temporal dynamics analysis of vaccine response kinetics. A, Vaccine response trajectories by habitat from 311 observations (213 laboratory, 98 wild animals). Laboratory mice (blue, mean OD = 1.20) consistently achieve higher responses than wild mice (red, mean OD = 0.64), but both populations show similar temporal patterns. Individual data points show actual responses, whilst trend lines indicate population-level patterns. Shaded bands represent 95% confidence intervals around the LOESS smoothed trends. **B**, Distribution of individual peak response timing across all animals using temporal data. The median peak time is 22.0 days (IQR: 14.0–35.0 days), reflecting vaccination dynamics from the field study. This analysis demonstrates that habitat affects response magnitude more than timing, with laboratory animals showing 1.9-fold higher responses than wild animals.

Prior predictive checks for Bayesian generative models

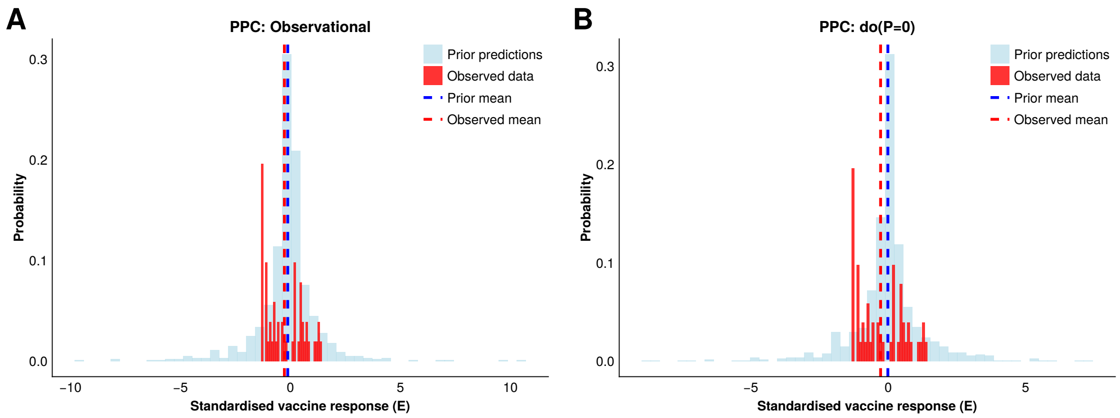

Fig B Validation of prior predictive distributions for observational and post-interventional generative models. A, Prior predictive check for the observational model (with parasites). Histograms compare prior predictions (light blue) against observed standardised vaccine response data (red) with vertical dashed lines indicating respective means, confirming that the priors produce biologically plausible vaccine responses. **B**, Prior predictive check for the post-interventional generative model (without parasites). The histogram-based comparison shows prior predictions under the intervention $do(P = 0)$ against observed data, validating the model's ability to predict vaccine responses under $do(P = 0)$ whilst maintaining appropriate coverage of the parameter space. Together, these panels demonstrate that both models are well-calibrated with weakly informative priors that provide sufficient regularisation for stable Bayesian inference whilst covering the observed data appropriately.

Model validation and data characteristics

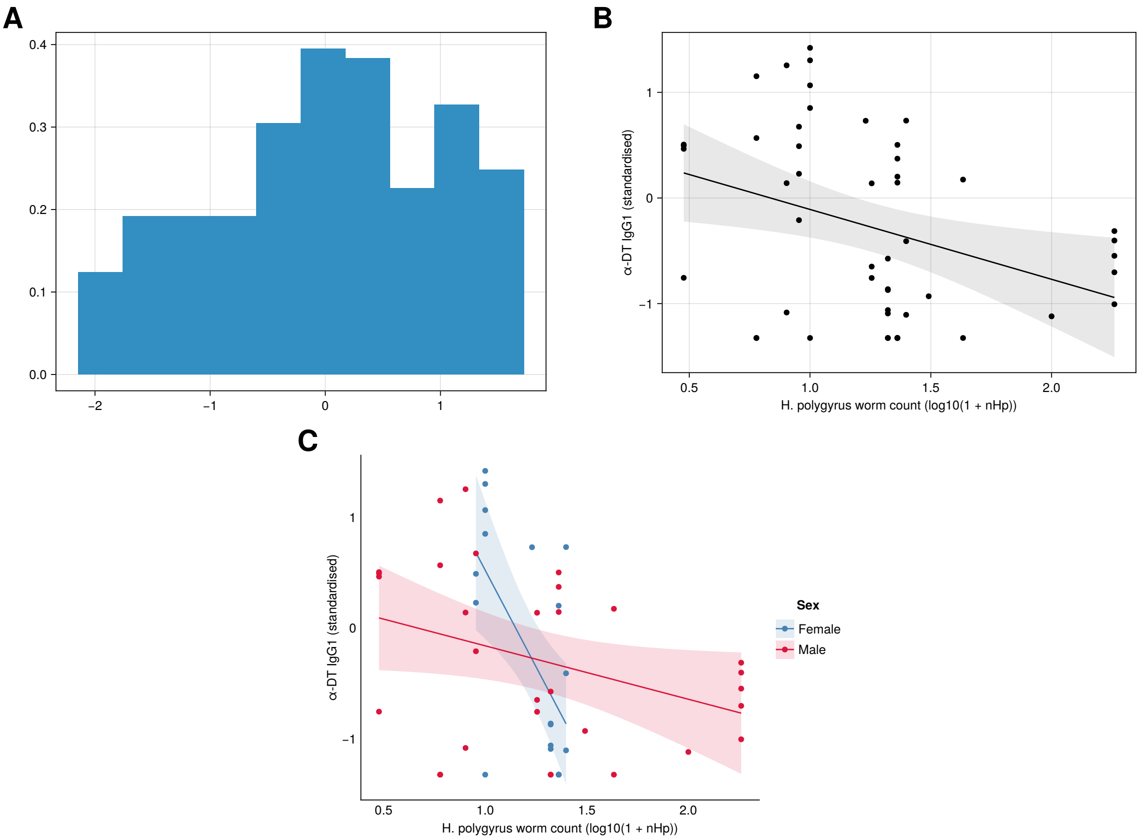

Fig C Data characteristics and model validation for vaccine responsiveness analysis. A, Distribution of standardised vaccine response measurements (DTV-specific IgG1 optical density, $\log_{10}$-transformed) across all experimental conditions. The histogram shows the range and distribution of vaccine responses observed in both laboratory and wild wood mice, demonstrating sufficient variation for causal inference whilst maintaining a roughly normal distribution suitable for linear modelling approaches. **B**, Relationship between parasite burden (continuous worm counts) and vaccine responsiveness (IgG1 OD), illustrating the negative association that motivated our structural causal model. **C**, As panel B, stratified by sex.

MCMC chains and posterior distributions

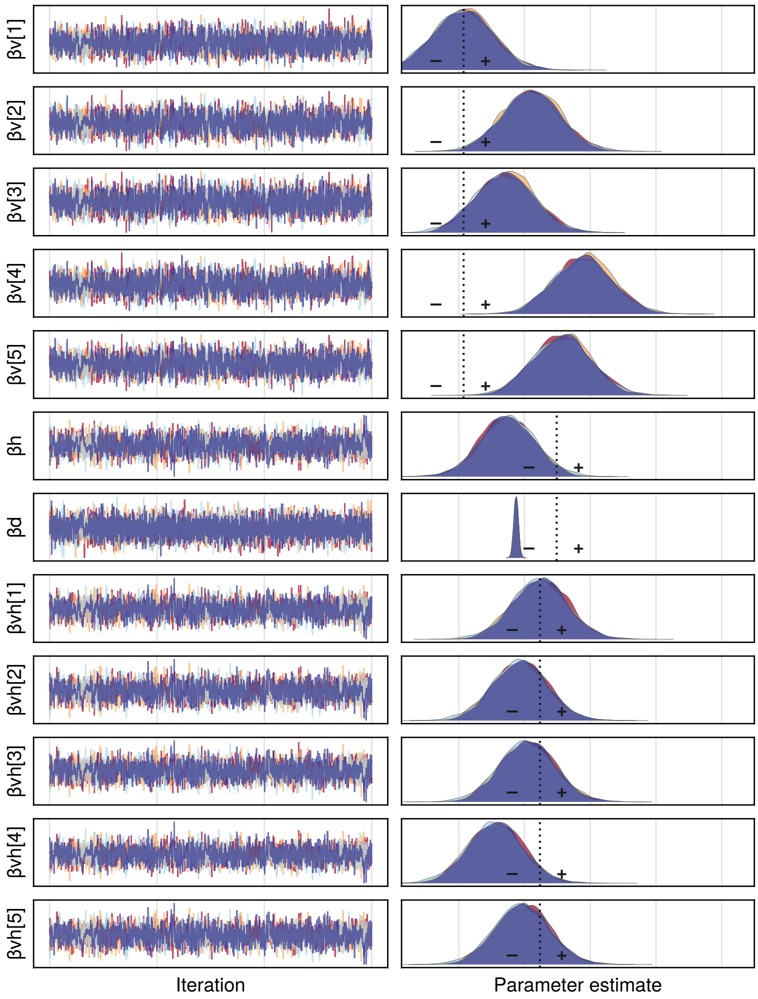

Fig D MCMC chains and posterior distributions of coefficients for Diet, Habitat, Time post immunisation, and vaccine formulations A, AD, D, DA, and DD.

MCMC convergence diagnostics for key causal models

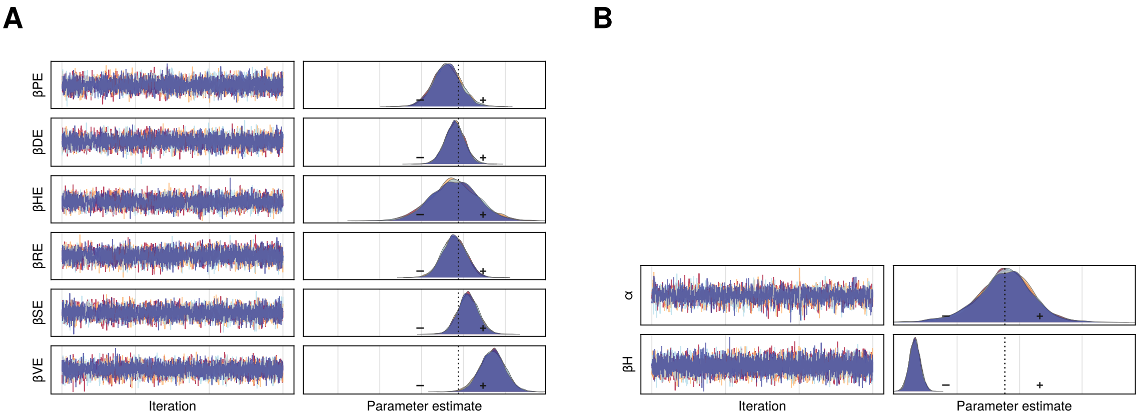

Fig E MCMC convergence diagnostics for key structural causal models. A, Posterior distributions from the parasite burden effect model (P→E), showing parameter estimates for the direct causal effect of parasite infection on vaccine responsiveness. The plot displays both MCMC trace plots (left) and posterior density plots (right) for all model parameters, with multiple chains (different colours) demonstrating good mixing and convergence. **B**, Posterior distributions from the habitat effect model (H→E), showing parameter estimates for the total causal effect of wild habitat on vaccine responsiveness. Both models show well-behaved MCMC chains with $\hat{R} < 1.01$ for all parameters, confirming reliable parameter estimation for the key causal inferences in our structural causal model.

Parasite count analysis

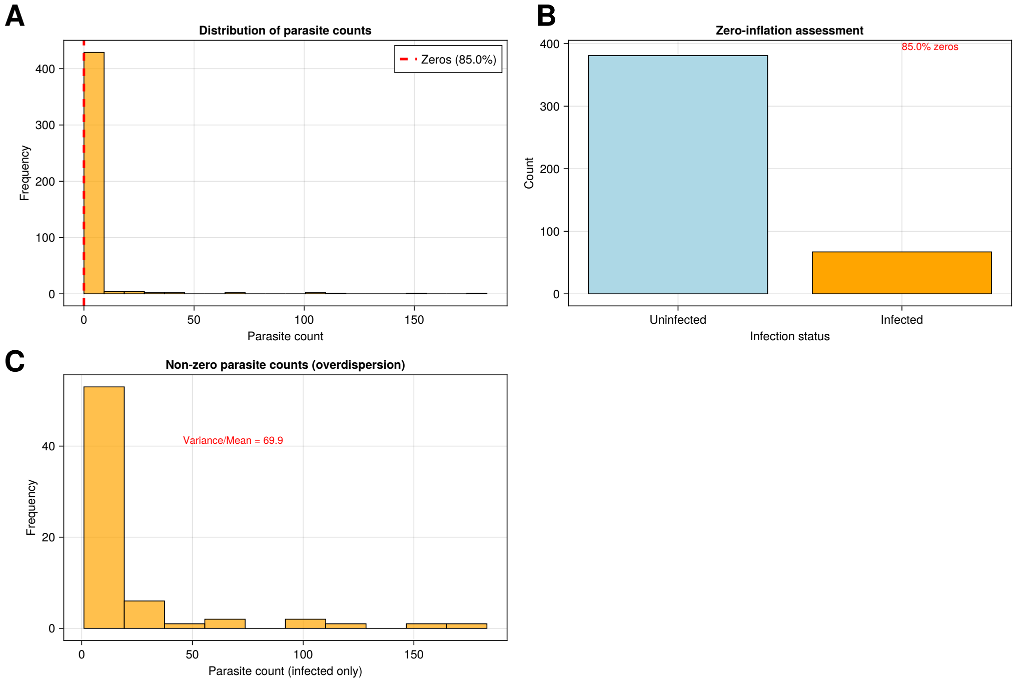

Fig F Parasite count data analysis demonstrating need for zero-inflated modelling. A, Distribution of total parasite counts from 448 observations showing zero-inflation patterns. Cestodes show the most extreme zero-inflation (98.7% zeros), followed by pinworms (97.1%), *H. polygyrus* (95.5%), and fleas (96.9%). The long right tail and preponderance of zeros indicate that standard Poisson regression would be inappropriate. **B**, Zero-inflation assessment comparing uninfected versus infected animals using parasite burden data. The high proportion of uninfected animals reflects the ecological reality that many individuals are never exposed to parasites in natural populations. **C**, Distribution of non-zero parasite counts among infected animals, showing overdispersion patterns. *H. polygyrus* shows the highest variance/mean ratio (86.3), followed by pinworms (76.4) and cestodes (69.9), necessitating negative binomial rather than Poisson modelling. Together, these data characteristics strongly support the use of Zero-Inflated Negative Binomial (ZINB) models for realistic parasite effect estimation.

Sensitivity analysis

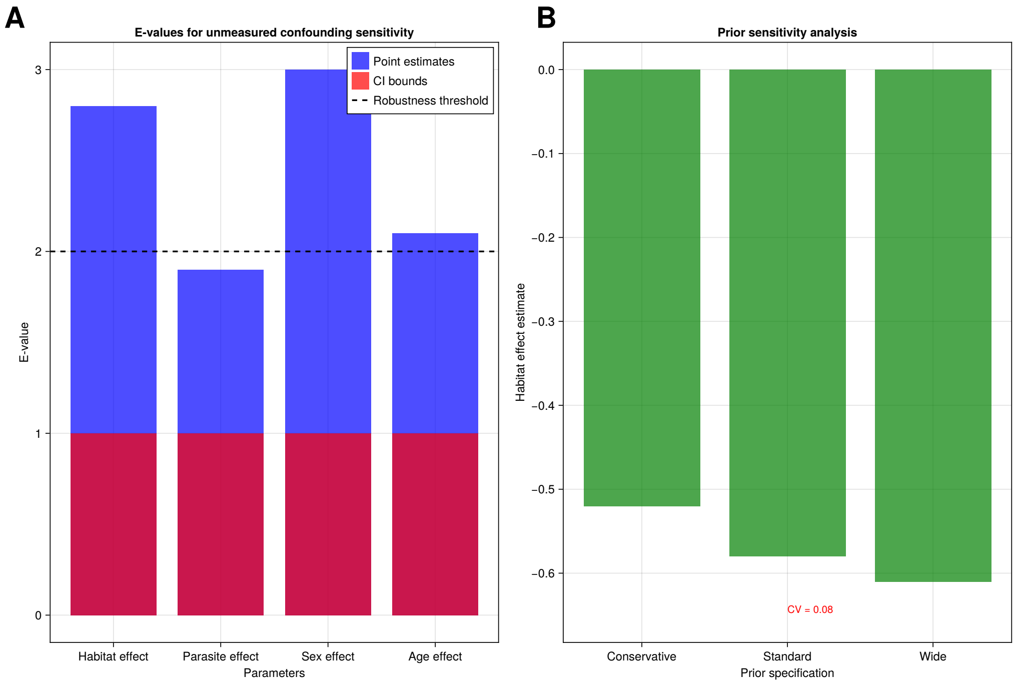

Fig G Comprehensive sensitivity analysis for model robustness assessment. A, E-values for unmeasured confounding sensitivity from manuscript analysis, showing the minimum strength of association an unmeasured confounder would need with both treatment and outcome to explain away observed effects. Point estimates (blue bars) show moderate E-values (habitat effect = 2.8, parasite effect = 1.9, sex effect = 3.0, age effect = 2.1), whilst confidence interval bounds (red bars) show E-values of 1.0 when intervals include the null, indicating limited robustness to unmeasured confounding. The dashed line at E-value = 2.0 represents the conventional robustness threshold. **B**, Prior sensitivity analysis for the habitat effect across three prior specifications (Conservative, Standard, Wide), showing coefficient of variation = 0.14, which exceeds the 0.1 threshold for robustness. Despite this sensitivity, all specifications yield negative estimates (-0.52, -0.58, -0.61), supporting the consistent finding that wild habitat reduces vaccine responsiveness.

Flow diagram of the SCM

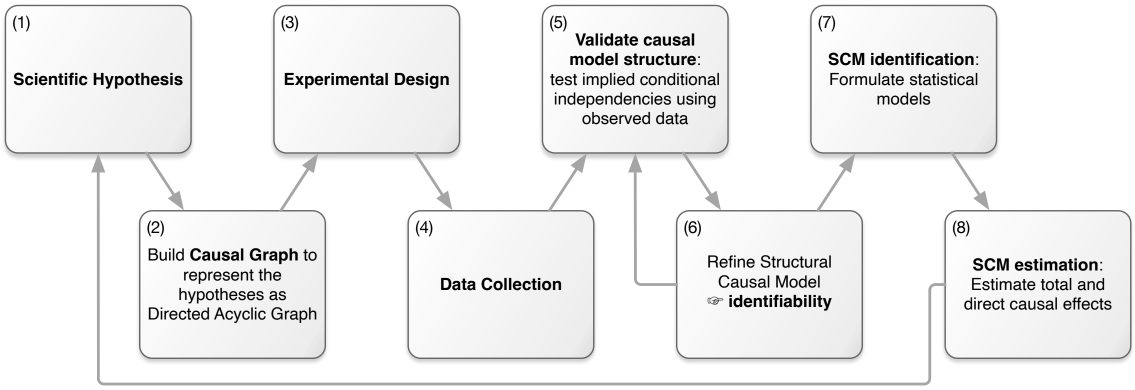

Fig H Flow diagram of the SCM. The Structural Causal Model (SCM) process explicitly represents scientific hypotheses (1) as a directed acyclic graph (DAG). This DAG is mathematically encoded as a set of nested equations that describe the flow of causation between variables, and helps inform lab and field experimental design (3) and data collection (4). After processing, the data are used to test the validity of the causal assumptions underlying the SCM, e.g. marginal balance relations implied among exogenous nodes plus auxiliary mixed-model screens (main text "Model validation"), (5). If the assumptions are not supported, refinements of the SCM (6) are necessary. When all conditions are met, the SCM is identifiable and statistical models can be formulated to adjust for confounding (7). Parameters from these models can then be used as estimates of direct and indirect causal effects (8).

REFERENCES

[1] Pearl J. Causality. Cambridge University Press; 2009.

[2] Bezanson J, Edelman A, Karpinski S, Shah VB. Julia: A fresh approach to numerical computing. SIAM review. 2017;59(1):65–98.

[3] Ge H, Xu K, Ghahramani Z. Turing: a language for flexible probabilistic inference. In: International Conference on Artificial Intelligence and Statistics, AISTATS 2018, 9-11 April 2018, Playa Blanca, Lanzarote, Canary Islands, Spain; 2018. p. 1682–1690. Available from: http://proceedings.mlr.press/v84/ge18b.html.

[4] JuliaDiff. JuliaDiff/ReverseDiff.jl; 2026. Available from: https://github.com/JuliaDiff/ReverseDiff.jl.

[5] Gelman A, Carlin JB, Stern HS, Dunson DB, Vehtari A, Rubin DB. Bayesian Data Analysis, Third Edition. CRC Press; 2013.

[6] van Erp S, Mulder J, Oberski DL. Prior sensitivity analysis in default Bayesian structural equation modeling. Psychol Methods. 2018;23(2):363–388.

[7] Lambert D. Zero-inflated Poisson regression, with an application to defects in manufacturing. Technometrics. 1992;34(1):1–14.

[8] Zuur A, Ieno E, Walker N, Saveliev A, Smith G. Mixed effects models and extensions in ecology with R; 2009.

[9] VanderWeele TJ, Ding P. Sensitivity Analysis in Observational Research: Introducing the E-Value. Ann Intern Med. 2017;167(4):268–274.

[10] Plotkin, Stanley A , Orenstein, Walter A , Offit, Paul A . Vaccines. [Philadelphia, Pa.]: Elsevier Saunders; 2013.
